# Specialized fibroblast differentiated states underlie scar formation in the infarcted mouse heart

**DOI:** 10.1172/JCI98215

**Published:** 2018-04-16

**Authors:** Xing Fu, Hadi Khalil, Onur Kanisicak, Justin G. Boyer, Ronald J. Vagnozzi, Bryan D. Maliken, Michelle A. Sargent, Vikram Prasad, Iñigo Valiente-Alandi, Burns C. Blaxall, Jeffery D. Molkentin

**Affiliations:** 1 Cincinnati Children’s Hospital Medical Center (CCHMC), Department of Pediatrics, University of Cincinnati, Cincinnati, Ohio, USA.; 2 AgCenter, School of Animal Sciences, Louisiana State University, Baton Rouge, Louisiana, USA.; 3 CCHMC, Howard Hughes Medical Institute, Cincinnati, Ohio, USA.

**Keywords:** Cardiology, Cardiovascular disease

## Abstract

Fibroblasts are a dynamic cell type that achieve selective differentiated states to mediate acute wound healing and long-term tissue remodeling with scarring. With myocardial infarction injury, cardiomyocytes are replaced by secreted extracellular matrix proteins produced by proliferating and differentiating fibroblasts. Here, we employed 3 different mouse lineage-tracing models and stage-specific gene profiling to phenotypically analyze and classify resident cardiac fibroblast dynamics during myocardial infarction injury and stable scar formation. Fibroblasts were activated and highly proliferative, reaching a maximum rate within 2 to 4 days after infarction injury, at which point they expanded 3.5-fold and were maintained long term. By 3 to 7 days, these cells differentiated into myofibroblasts that secreted abundant extracellular matrix proteins and expressed smooth muscle α-actin to structurally support the necrotic area. By 7 to 10 days, myofibroblasts lost proliferative ability and smooth muscle α-actin expression as the collagen-containing extracellular matrix and scar fully matured. However, these same lineage-traced initial fibroblasts persisted within the scar, achieving a new molecular and stable differentiated state referred to as a matrifibrocyte, which was also observed in the scars of human hearts. These cells express common and unique extracellular matrix and tendon genes that are more specialized to support the mature scar.

## Graphical abstract

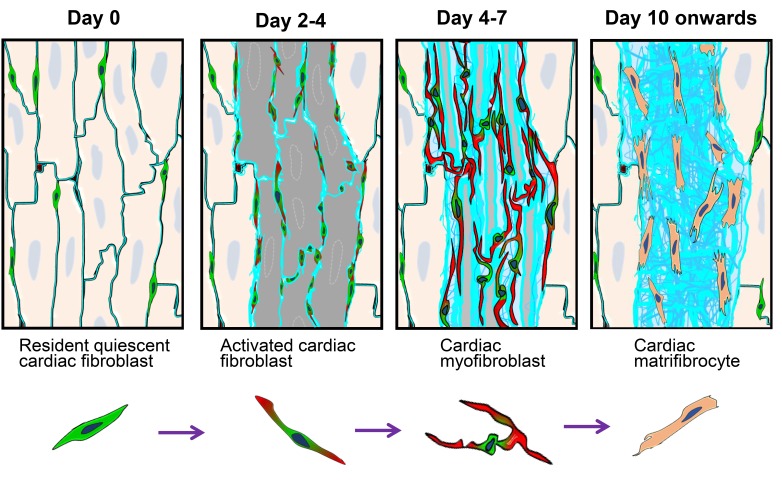

## Introduction

Fibroblasts are a unique cell type of mesenchymal origin that are present in essentially all tissues and organs, where they regulate extracellular matrix (ECM) production and acute wound healing (1–3). In addition to regulating local fibrosis and ECM production, fibroblasts also secrete growth factors to support tissue regeneration in coordination with inflammatory cells (4–6). In response to injury, tissue stretching, and cytokines such as TGF-β, fibroblasts become activated and then differentiate into a cell type referred to as a myofibroblast. Once induced, myofibroblasts produce and secrete greater levels of ECM proteins, including multiple types of collagen, and they express contractile genes, such as smooth muscle α-actin (αSMA), that underlie their ability to contract and close a wounded area (7, 8). In addition to forming myofibroblasts, unactivated tissue-resident fibroblasts have been reported to differentiate into endothelial cells and perhaps even tissue parenchymal cells at a low frequency (9–11), suggesting that these cells have some degree of multilineage competency.

In most tissues or organs, injury-induced fibrosis is a transient process that typically regresses once the healing response is finalized (4, 12–14). However, the heart is perhaps one of the more unique organs with respect to the fibrotic response and wound healing, as this tissue is not inherently regenerative and a stable scar must quickly form to prevent ventricular wall rupture. Heart disease remains the number one cause of mortality in the Western world, with atherosclerosis and subsequent myocardial infarction (MI) injury representing the most lethal subset of disease (15, 16). Acute MI causes rapid necrotic and apoptotic loss of cardiomyocytes within the ischemic region, and thereafter, the activity of fibroblasts becomes critical in buttressing the ventricular wall as the fibrotic scar forms over several days (17–20). Previous work has shown that αSMA^+^ myofibroblasts are present within the infarct area after acute MI injury (21). While multiple cell types have been suggested as generating myofibroblasts in the diseased heart, more recent genetic lineage–tracing analysis has shown that tissue-resident fibroblasts of developmental epicardial origin, which make up approximately 12% of all the cells in the adult rodent heart, are the overwhelming source for myofibroblast generation (22–25).

Despite the critical role that fibroblasts play in the diseased heart, the field has lacked an in-depth understanding of this cell type and its dynamics with MI injury. However, with the recent advent of multiple genetic lineage–tracing mouse models, it is now possible to dissect these cells and determine their phenotypic and functional characteristics during MI injury. Here, we used fibroblast stage-specific lineage tracing along with mechanistic analysis of MI-injured mice and mRNA expression profiling to annotate the stable differentiated states and dynamics of fibroblasts in the heart, which defines a fibroblast state referred to as the matrifibrocyte that resides within the mature scar.

## Results

### Cardiac fibroblast proliferation following MI.

**Figure 1:**
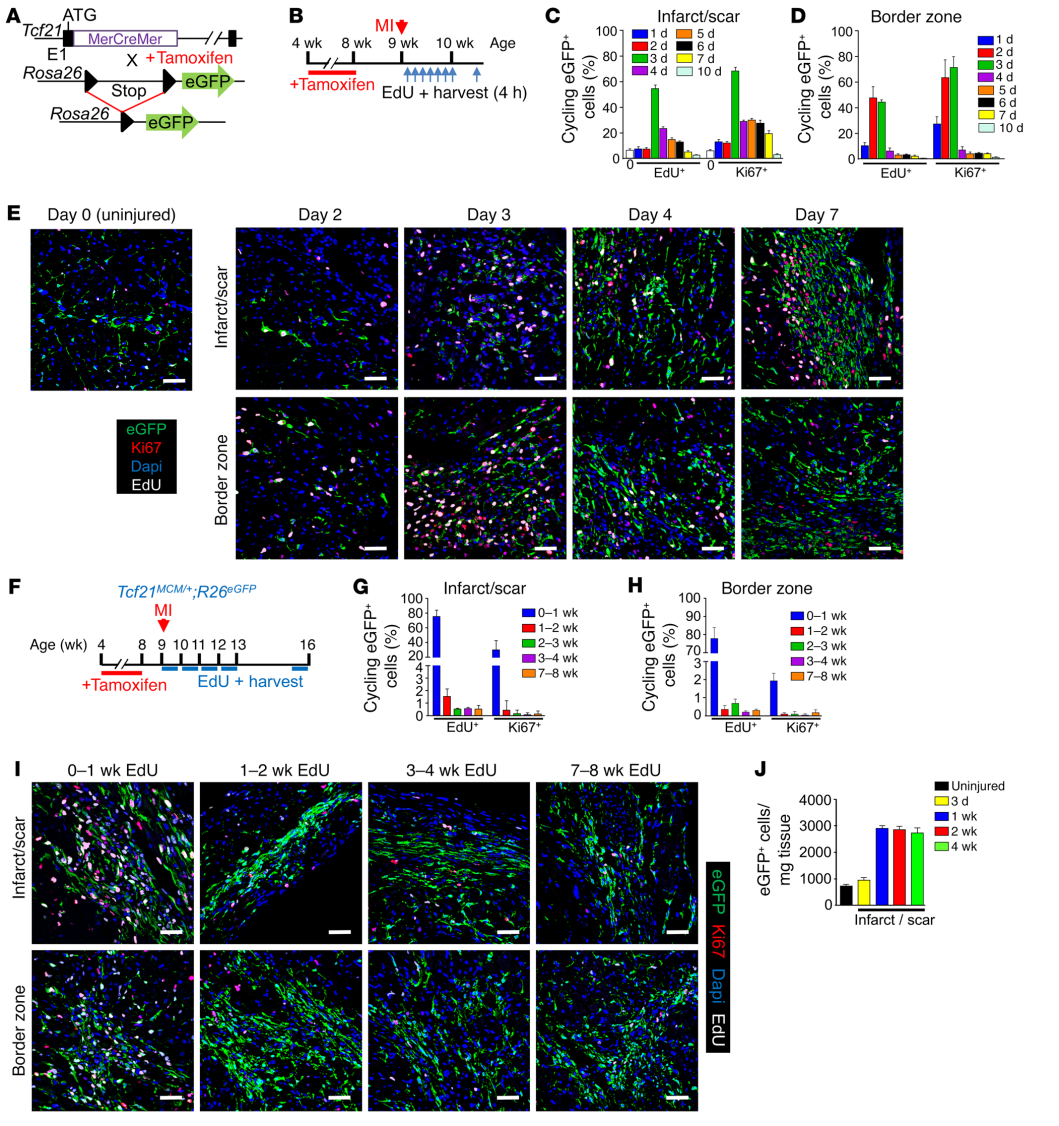
Proliferation of *Tcf21* lineage–traced fibroblasts after MI. (**A**) Schematic of the *Tcf21* genetic locus with a tamoxifen-regulated MerCreMer cDNA cassette inserted into exon 1 (E1).The MerCreMer-containing *Tcf21* locus was introduced into *R26^EGFP^* mice containing a loxP site–flanked stop cassette upstream of *EGFP* to allow for Cre-dependent lineage tracing. (**B**) Experimental scheme whereby *Tcf21^MCM/+^*;*R26^EGFP^* mice were given tamoxifen for 4 weeks and rested for 1 week before MI surgery. Mice were treated with a single EdU injection at the indicated time points after MI, and hearts were harvested 4 hours after each EdU injection for IHC analysis. (**C**–**E**) Quantification of EdU^+^ (white) and Ki67^+^ (red) *Tcf21* lineage–traced (EGFP^+^) fibroblasts (green) in infarct region (**C**) and border zone (**D**) after a single EdU injection at the indicated time points after MI by IHC and representative IHC images. The white bar in **C** represents a 0 time point. (**E**) Nuclei are shown with DAPI (blue); these same images are shown in Supplemental Figure 1C in a larger temporal array. (**F**) Experimental scheme of tamoxifen treatment of *Tcf21^MCM/+^*;*R26^EGFP^* mice before MI surgery and 7 daily EdU injections during indicated time periods after MI. Hearts were harvested 4 hours after the last EdU injection for IHC analysis. (**G**–**I**) Quantification of EdU^+^ (white) and Ki67^+^ (red) *Tcf21* lineage–traced fibroblasts (green) in the infarct region (**G**) and border zone (**H**) after 7 daily EdU injections during the indicated time periods after MI by IHC and representative IHC images (**I**). Nuclei are shown with DAPI (blue). (**J**) Quantification of *Tcf21* lineage–traced fibroblasts in the infarct region at the indicated time points by FACS. Density of cells is presented as the number of cells per mg of infarct tissue. (**C**, **D**, **G**, **H**, and **J**) Data are shown as mean ± SD (*n* = 3). **E** and **I** show representative images from 3 separate hearts analyzed. Scale bars: 20 μm.

To examine the proliferation dynamics of cardiac fibroblasts in response to MI, we employed a lineage tracking system with *Tcf21^MCM/+^*;*R26^EGFP^* mice that expresses the MerCreMer tamoxifen-regulated protein under the control of the transcription factor 21 (*Tcf21*) gene locus (26) in conjunction with a loxP site–dependent EGFP reporter that resides within the ubiquitous *Rosa26* gene locus (Figure 1A and Supplemental Figure 1A; supplemental material available online with this article; https://doi.org/10.1172/JCI98215DS1). We previously showed that this lineage-tracing reporter system identifies the vast majority of tissue-resident cardiac fibroblasts in the uninjured and injured adult mouse heart (23). Here, 4-week-old *Tcf21^MCM/+^*;*R26^EGFP^* mice were put on tamoxifen diet for 4 weeks to specifically label all resident fibroblasts; thereafter, the mice received an MI surgical procedure at 9 weeks of age (Figure 1B and Supplemental Figure 1B). To quantify proliferating cells, a single dose of 5-ethynyl-2′-deoxyuridine (EdU) was given to mice for 4 hours at multiple selected time points after MI injury, followed by heart removal and IHC examination of EdU and Ki67 staining (Figure 1B and Supplemental Figure 1B). The data show that 1 to 2 days after MI injury, *Tcf21* lineage–traced fibroblasts did not proliferate in the infarct region of the heart, although by day 3, there was a robust induction of proliferation (Figure 1, C and E, and Supplemental Figure 1C). The border zone region showed proliferation of *Tcf21* lineage–traced fibroblasts 1 day earlier, likely because most of the fibroblasts died within the infarct region and 1 additional day was needed for these cells to move into the infarct from the border zone (Figure 1, D and E). In the infarct region, fibroblasts continued to proliferate through day 7, although proliferation in the border zone was extinguished after day 3 (Figure 1, C–E, and Supplemental Figure 1C). These data indicate that resident cardiac fibroblasts proliferate robustly within 2 days of MI injury, reaching a peak by 2 to 4 days, with reduced but detectable proliferation thereafter through day 7 within the infarct.

We also analyzed fibroblast proliferation over longer periods of time using 7 consecutive days of EdU injections in mice, followed by harvesting hearts every week for 8 additional weeks (Figure 1F). The data show that essentially all fibroblast proliferation occurs within the first week of MI injury in the infarct and border zone regions (Figure 1, G–I). Total fibroblast content within the infarct region also expands by approximately 3.5-fold versus the uninjured heart, and this expansion in fibroblast content remains stable over 4 weeks, suggesting that newly made fibroblasts persist within the scar region long term (Figure 1J). As further quantitation, FACS analysis of EdU-labeled *Tcf21* lineage–traced fibroblasts within the infarct region was also performed. As with the IHC analysis, FACS analysis showed a peak of cardiac fibroblast proliferation at day 3 after MI and lesser but detectable proliferation at day 7 compared with no proliferation at day 28 (Supplemental Figure 1E). Finally, lineage-traced fibroblast proliferation rates were minimal or nonexistent within the uninjured regions of these same hearts, such as the septum and right ventricle, at all time points up to 4 weeks (data not shown).

The results presented above employed *Tcf21*-based lineage tracing to identify cardiac fibroblasts after MI injury. We previously demonstrated that the periostin (*Postn*) genetic locus containing the MerCreMer cDNA linage traces essentially all activated cardiac fibroblasts in the heart, the overwhelming majority of which come from Tcf21^+^ resident fibroblasts (23). Here, we used a knockin allele of *Postn* containing the β-galactosidase (LacZ) expression cassette to track current expression from that locus. Importantly, these same *Tcf21*-traced cardiac fibroblasts coexpressed the *Postn* locus 4 days after MI injury within the heart (Supplemental Figure 2A). Using *Postn^MCM/+^*;*R26^EGFP^* mice, we observed similar proliferation dynamics in *Postn* lineage–traced activated fibroblasts over 4 weeks time after MI injury as compared with *Tcf21* lineage–traced cells (Supplemental Figure 2, B–E), which further validates the conclusion that activated fibroblasts can proliferate in the heart with MI injury.

### Differentiation of cardiac fibroblasts to myofibroblasts after MI.

**Figure 2:**
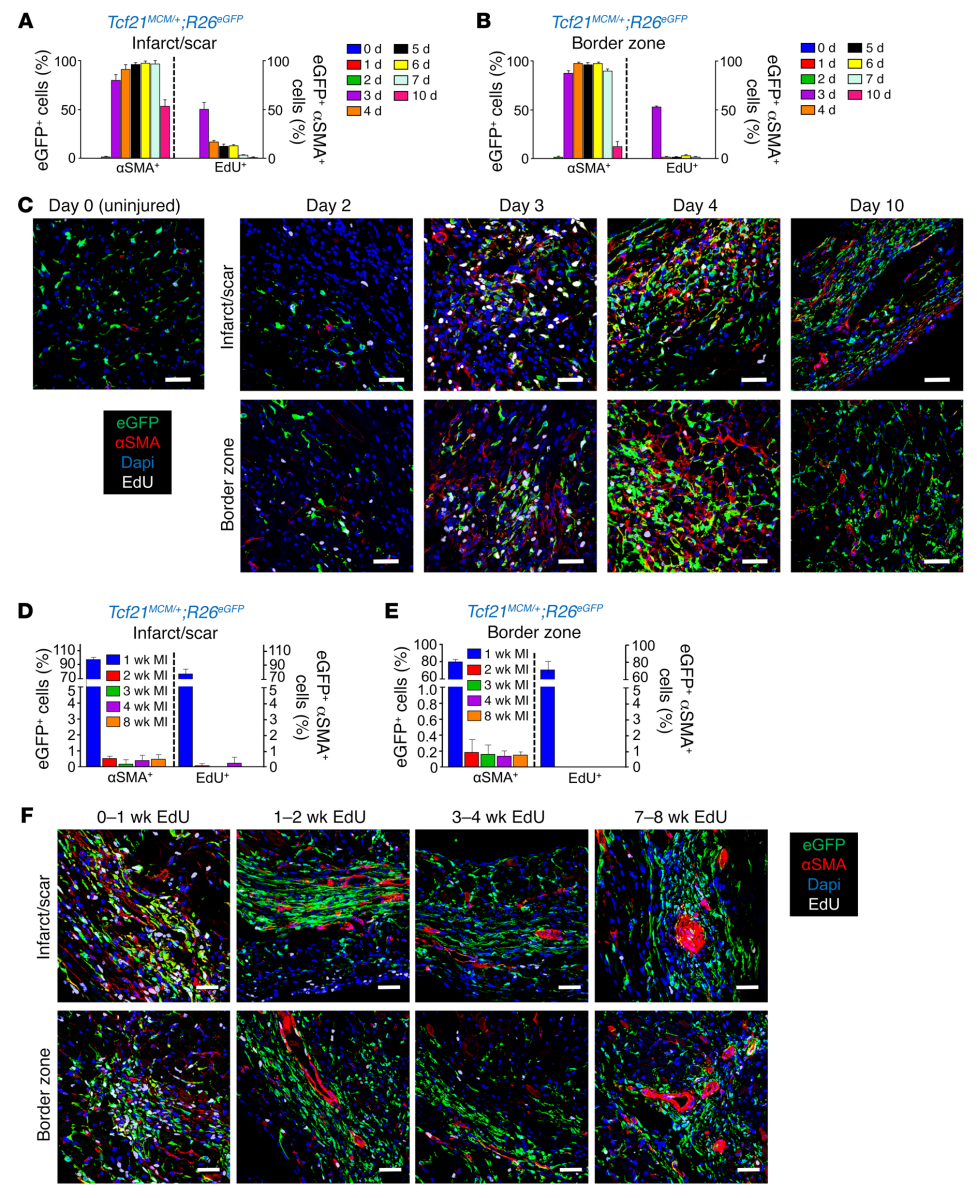
Differentiation of *Tcf21* lineage–traced fibroblasts into myofibroblasts after MI. (**A** and **B**) Quantification of αSMA^+^ and EdU^+^ *Tcf21* lineage–traced fibroblasts in the MI region (**A**) and border zone (**B**) after a single EdU injection at the indicated time points after MI by IHC. (**C**) Representative IHC images showing αSMA^+^ (red) and EdU^+^ (white) *Tcf21* lineage–traced (EGFP^+^) fibroblasts quantified in **A** and **B**. Nuclei are shown with DAPI (blue). These same images from **C** are shown in Supplemental Figure 1D in a larger temporal array. (**D**–**F**) Quantification of αSMA^+^ (red) and EdU^+^ (white) *Tcf21* lineage–traced fibroblasts in the MI region (**D**) and border zone (**E**) after 7 daily EdU injections during the indicated time periods after MI by IHC and representative IHC images (**F**). Nuclei are shown with DAPI (blue). (**A**, **B**, **D** and **E**) Data are shown as mean ± SD (*n* = 3). **C** and **F** show representative images from 3 separate hearts analyzed. Scale bars: 20 μm.

Myofibroblasts are traditionally identified by newly acquired expression of the *Acta2* gene (αSMA protein), and these cells are known to reside within the scar region of the infarcted myocardium (21, 27), although we know little else of their dynamics. Here, *Tcf21^MCM/+^*;*R26^EGFP^* mice were given tamoxifen for 1 month to prelabel quiescent resident cardiac fibroblasts so that proliferation could be correlated with αSMA expression by IHC after MI injury. Single EdU injections were again performed each day over 10 days, and mice were harvested 4 hours after each injection, as shown in Figure 1B. Myofibroblast formation and αSMA expression were first observed in *Tcf21* lineage–traced cardiac fibroblasts in both the infarcted area and border zone 3 days after MI injury, coinciding with peak proliferation (Figure 2, A–C). While proliferation was mostly extinguished by day 7 in the infarct area, αSMA expression remained high through 7 days and was even partially detectable by day 10 in these fibroblasts, but then expression was absent by day 14 (Figure 2, A–C, and Supplemental Figure 1D). These results indicate that myofibroblasts in the MI-injured heart can proliferate, with a peak effect at day 3. These results also suggest that myofibroblasts are not a long-lasting differentiated state, as by days 7 to 10 after MI, expression of αSMA was reduced, then entirely lost in the *Tcf21* lineage–traced fibroblasts.

*Tcf21^MCM/+^*;*R26^EGFP^* mice were again subjected to 7 consecutive days of EdU injections, followed by harvesting hearts every week for 4 to 8 additional weeks, followed by αSMA IHC analysis (Figure 1F). Essentially all of the proliferating αSMA-positive *Tcf21* lineage–traced cells were identified in the first week in the border zone and infarct region, with loss of proliferation and current αSMA expression by 2 weeks after MI, although all of the *Tcf21* lineage–traced cells persisted onwards of 8 weeks (Figure 2, D–F). Two weeks after MI, αSMA-positive cells within the border zone and infarct region were restricted to vessels with vascular smooth muscle (Figure 2F). Quantitation by FACS analysis also showed αSMA expression in *Tcf21* lineage–traced fibroblasts at days 3 and 7 after MI, but not at 28 days (Supplemental Figure 1E). Nearly identical results were observed with *Postn^MCM/+^*;*R26^EGFP^* mice after MI as a means of lineage-tracing activated fibroblasts (Supplemental Figure 2F). Approximately 90% of *Postn* lineage–traced activated fibroblasts were αSMA expressing at days 4 to 7 after MI, but by 2 weeks and afterwards, proliferation and αSMA expression were lost from these fibroblasts (Supplemental Figure 2, G and H). Taken together, these results indicate that activated fibroblasts and myofibroblasts in the heart proliferate during the first 7 days of MI injury, but then progress to an alternate differentiated state that loses proliferation capacity and αSMA expression.

### Apoptosis and turnover of fibroblasts in the infarcted myocardium.

**Figure 3:**
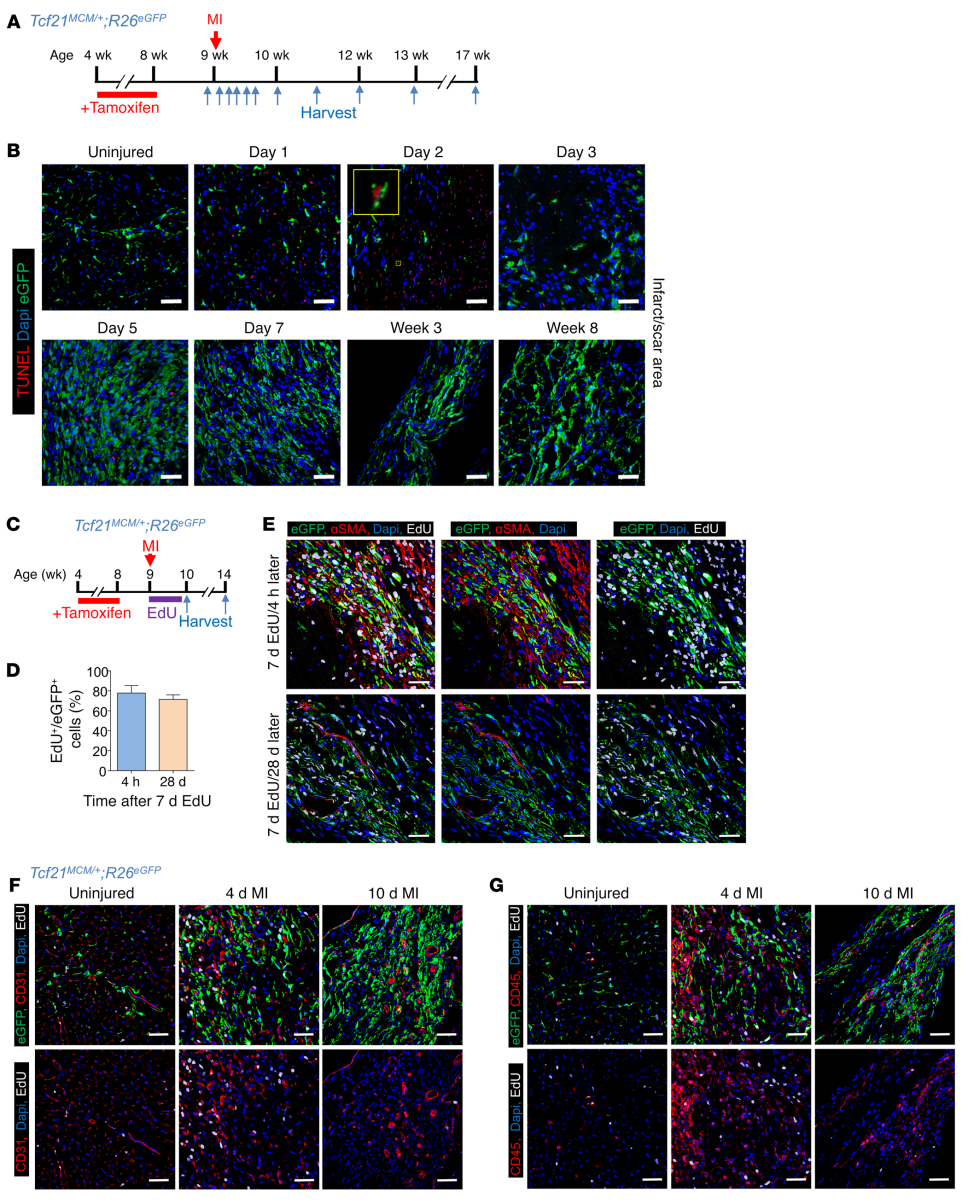
Apoptosis and turnover of *Tcf21* lineage–traced fibroblasts after MI. (**A**) Experimental scheme of tamoxifen treatment of *Tcf21^MCM/+^*;*R26^EGFP^* mice before MI. Hearts were harvested at the indicated time points after MI for TUNEL staining. (**B**) Representative TUNEL staining (red) images from 3 separate hearts analyzed showing apoptotic *Tcf21* lineage–traced (EGFP^+^) fibroblasts after MI. Nuclei are shown with DAPI (blue). The inset shows a higher magnification (×8) image of a TUNEL^+^ EGFP^+^ cell. (**C**) Experimental scheme of tamoxifen treatment of *Tcf21^MCM/+^*;*R26^EGFP^* mice before MI followed by 7 daily EdU injections during the first week after MI. Hearts were then harvested 4 hours and 28 days after the last EdU injection for IHC analysis. (**D** and **E**) Quantification (**D**) of EdU^+^ (white) *Tcf21* lineage–traced (EGFP^+^) fibroblasts in the infarcted area at 4 hours and 28 days after the last injection of 7 daily EdU injections. (**E**) Nuclei are shown with DAPI (blue), and αSMA (red) was stained to show myofibroblast identity. Data are shown as mean ± SD (*n* = 3 hearts analyzed). Two-tailed *t* test showed no significance. For **E**, representative IHC images are shown from 3 separate hearts analyzed. (**F**) Representative IHC images from 3 separate hearts analyzed showing EdU^+^ (white) CD31^+^ endothelial cells (red) versus *Tcf21* lineage–traced fibroblasts (EGFP^+^) in hearts before MI and within infarct region at day 4 and day 10 after MI 4 hours after a single EdU injection. (**G**) Representative IHC images from 3 hearts analyzed showing EdU^+^ (white) CD45^+^ leukocytes (red) versus *Tcf21* lineage–traced fibroblasts (EGFP^+^) in hearts before MI and within infarct region at day 4 and day 10 after MI 4 hours after a single EdU injection. Scale bars: 20 μm.

We observed that *Tcf21* lineage–traced fibroblasts were largely lost at day 2 from the region of the heart that suffered infarction injury, but by days 3 to 4, these cells rapidly rebounded and expanded throughout the injured area, where they persisted long term (Figure 1E and Supplemental Figure 1C). To examine cell death in contributing to the dynamics of fibroblasts within the MI region, we performed TUNEL on hearts from *Tcf21^MCM/+^*;*R26^EGFP^* mice before and after MI (Figure 3A). Apoptotic nuclei within the infarcted area of the heart were observed at day 1 after MI; this peaked at day 2 and then became almost undetectable by day 7 and onwards (Figure 3B). Importantly, a substantial number of *Tcf21* lineage–traced fibroblasts were TUNEL positive at day 2 after MI, but not at day 3 (Figure 3B). At day 2 after MI, most of the TUNEL^+^ cardiac fibroblasts had condensed nuclei and showed a scattered and fragmented EGFP signal (Figure 3B). We also quantified total fibroblast numbers within the infarct 4 hours or 28 days after a wave of EdU labeling during only the first 7 days after MI (Figure 3C). We observed that roughly 75% of *Tcf21* lineage–traced (EGFP^+^) fibroblasts were labeled by EdU over 7 days of injections after MI (Figure 3, D and E). However, if these same mice labeled with EdU over 7 days were instead harvested at day 28 after EdU injection, the same relative number of EGFP^+^ cells were EdU^+^, indicating that newly formed fibroblasts are stable and not turned over in the infarct region (Figure 3, D and E). Taken together, these results indicate that proliferating and newly activated cardiac fibroblasts and myofibroblasts are a robust cell type that persists in the MI-injured area.

Fibroblasts are known for their plasticity and potential ability to transdifferentiate into other cell types, such as immune cells or endothelial cells (9, 28). To determine whether newly generated fibroblasts in the infarcted heart have such an ability, hearts from the same *Tcf21^MCM/+^*;*R26^EGFP^* mice with lineage-traced fibroblasts were subjected to IHC for CD31 (endothelial) and CD45 (leukocytes). We were unable to detect events whereby a *Tcf21* lineage–traced fibroblast converted to a CD31- or CD45-expressing cell (Figure 3, F and G, and Supplemental Figure 3, A–C). Interestingly, continuous low-level proliferation of CD31- and CD45-expressing cells was observed throughout all stages that were analyzed after MI, indicating persistence of inflammation and angiogenesis (Figure 3, F and G, Supplemental Figure 3, B and C).

### Lineage tracing specifically for myofibroblasts in the heart.

**Figure 4:**
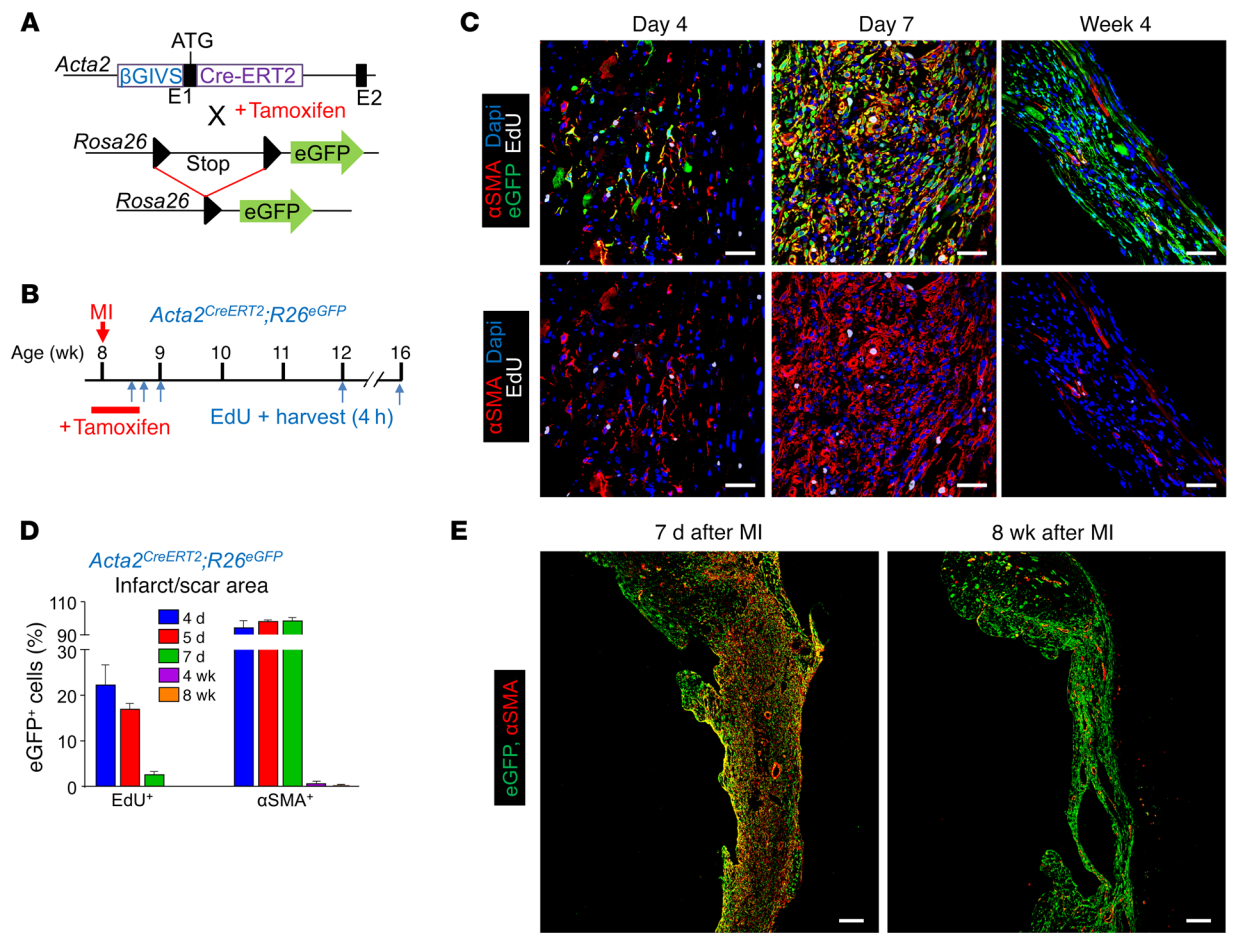
Lineage tracing of myofibroblasts in *Acta2^CreERT2^*;*R26^EGFP^* mice. (**A**) Schematic of the *Acta2* BAC with a tamoxifen-regulated CreERT2 cDNA cassette inserted into exon 1, and mice containing this transgene were crossed with *R26^EGFP^* reporter mice containing a loxP site–flanked stop cassette upstream of EGFP to allow for Cre-dependent lineage tracing. (**B**) Experimental scheme whereby *Acta2^CreERT2^*;*R26^EGFP^* mice were given tamoxifen through daily i.p. injections from day –1 to day 4 after MI. Mice were treated with a single EdU injection at the indicated time points after MI, and hearts were harvested 4 hours afterward for IHC analysis. (**C** and **D**) Representative IHC images from 3 hearts analyzed showing αSMA protein (red) and EdU^+^ (white) in αSMA lineage–traced (EGFP^+^) fibroblasts in the infarct region after a single EdU injection at the indicated time points after MI (**C**) and quantification (**D**). Nuclei are shown with DAPI (blue). Data are shown as mean ± SD (*n* = 3). Scale bars: 20 μm. (**E**) Representative IHC images from 3 separate hearts analyzed showing αSMA lineage–traced (EGFP^+^) fibroblasts and expression of αSMA protein (red) in the infarct region and border zone at 7 days and 8 weeks after MI. Nuclei are shown with DAPI (blue). Scale bars: 200 μm.

We also used *Acta2^CreERT2^*;*R26^EGFP^* mice to lineage trace myofibroblasts specifically. The *Acta2^CreERT2^* mice contain a bacterial artificial chromosome (BAC) transgene that expresses the tamoxifen-inducible Cre-ERT2 protein within a portion of the *Acta2* genomic locus (Figure 4A) (29). *Acta2^CreERT2^*;*R26^EGFP^* mice were given tamoxifen 1 day before and immediately after MI injury for 5 days total, after which EdU was given at select time points to measure proliferation (Figure 4B). Similarly to what occurred in *Postn^MCM/+^*;*R26^EGFP^* mice, *Acta2* lineage–traced myofibroblasts were first observed at day 4 after MI (Figure 4, C and D). Importantly, IHC for αSMA protein showed that essentially all *Acta2* lineage–traced fibroblasts were also positive for αSMA protein in the first 7 days, demonstrating the high fidelity of the *Acta2*^CreERT2^;*R26^EGFP^* genetic approach (Figure 4, C and D). However, by 4 and 8 weeks after MI injury, these *Acta2* lineage–traced fibroblasts persisted within the infarcted region of the heart despite the complete absence of αSMA protein expression, with only vascular smooth muscle cells within vessels showing expression (Figure 4, C and E). With respect to proliferation, some *Acta2* lineage–traced fibroblasts were EdU positive within the first 7 days after MI injury, but were refractory to cell-cycle activity thereafter (Figure 4, C and D). Again, we observed no evidence of transdifferentiation of *Acta2* lineage–traced myofibroblasts into endothelial cells or leukocytes based on CD31 and CD45 IHC (Supplemental Figure 3, D and E).

### Cardiac fibroblasts in the scar have a unique differentiated state.

**Figure 5:**
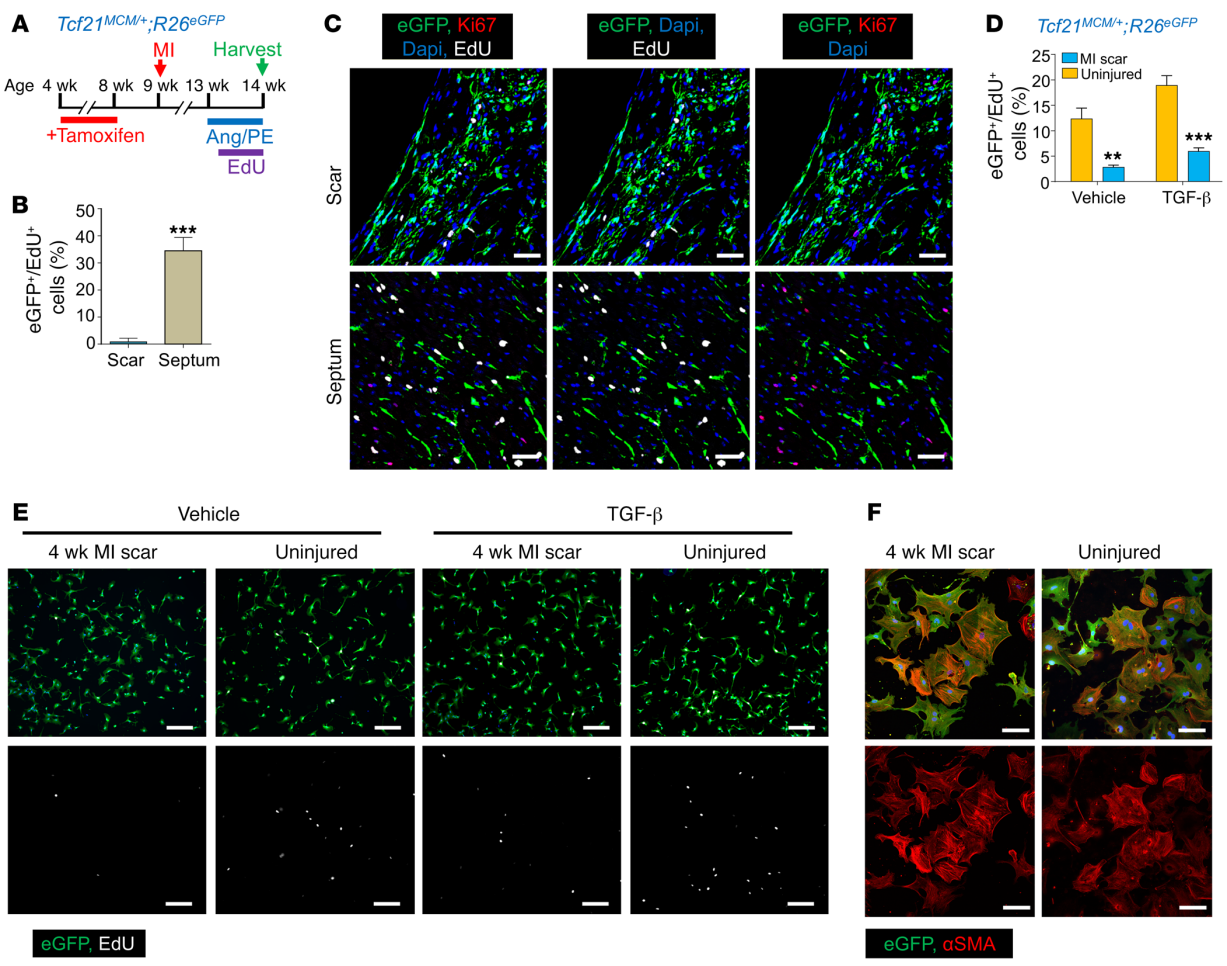
Proliferation potential of *Tcf21* lineage–traced fibroblasts residing in stable scar. (**A**) Experimental scheme whereby *Tcf21^MCM/+^*;*R26^EGFP^* mice previously treated with tamoxifen were subjected to MI and then treated with Ang II/PE through osmotic pump 4 weeks after MI. Mice were treated with EdU through daily i.p. injections for 6 days starting at day 2 after pump implantation, and hearts were harvested 4 hours after the last EdU injection for IHC analysis. (**B** and **C**) Quantification (**B**) and representative IHC images from 3 hearts analyzed (**C**) of EdU^+^ (white) and Ki67^+^ (red) *Tcf21* lineage–traced (EGFP^+^) fibroblasts in the infarct region and septum of hearts from *Tcf21^MCM/+^*;*R26^EGFP^* mice that received treatment as shown in **A**. Nuclei are shown with DAPI (blue). Scale bars: 20 μm. (**D** and **E**) Quantification (**D**) and representative immunocytochemistry from 3 separate experiments (**E**) of EdU^+^ (white) *Tcf21* lineage–traced (EGFP^+^) fibroblasts isolated from uninjured hearts and the infarct region of hearts 4 weeks later. EdU was given for 6 hours with and without TGF-β stimulation. Scale bars: 200 μm. (**F**) Representative immunocytochemistry images from 3 separate experiments showing αSMA stress fibers (red) in *Tcf21* lineage–traced fibroblasts isolated from uninjured hearts and the infarct region of hearts 4 weeks after MI. Cells were also treated with TGF-β for 3 days. Scale bars: 10 μm. (**B** and **D**) Data are shown as mean ± SD (*n* = 3). **P < 0.01, ***P < 0.0001, 2-tailed *t* test.

The lack of EdU uptake and loss of αSMA expression in fibroblasts residing within the relatively hypoxic and high-tension scar region of the heart suggested that these cells achieved an alternate differentiated state. To solidify these observations, we used a model in which 2 rounds of fibroblast proliferation were induced in the mouse heart. *Tcf21^MCM/+^*;*R26^EGFP^* mice were first treated with tamoxifen to label all fibroblasts with EGFP; then MI injury was performed, followed 4 weeks later by placement of s.c. osmotic pumps loaded with angiotensin II (Ang II) and phenylephrine (PE). Infusion of these 2 agonists is known to activate fibroblasts in the heart and cause proliferation with ensuing interstitial fibrosis (30, 31) (Figure 5A). Remarkably, the *Tcf21* lineage–traced fibroblasts within the MI scar region were refractory to subsequent Ang II/PE–induced EdU incorporation and proliferation, while resident fibroblasts in the remainder of the heart, such as the septum, readily proliferated (Figure 5, B and C). Analysis of Ki67 expression as an indicator of proliferation showed a profile similar to that of EdU (Figure 5C). We also sorted *Tcf21* lineage–traced (EGFP^+^) fibroblasts from the uninjured heart versus the infarct scar region 28 days after MI for assessment of proliferation in culture, with and without TGF-β stimulation. Under these conditions, 6 hours of EdU treatment labeled significantly more *Tcf21* lineage–traced fibroblasts isolated from the uninjured heart compared with the scarred region, with or without TGF-β stimulation (Figure 5, D and E). However, TGF-β treatment was equally efficient in promoting αSMA stress fiber formation in either population of isolated fibroblasts (Figure 5F). Taken together, these results suggest that fibroblasts within the infarcted scar region have unique properties that could represent a previously unappreciated differentiated state.

### Myofibroblasts stabilize the scar before the collagen scar forms.

**Figure 6:**
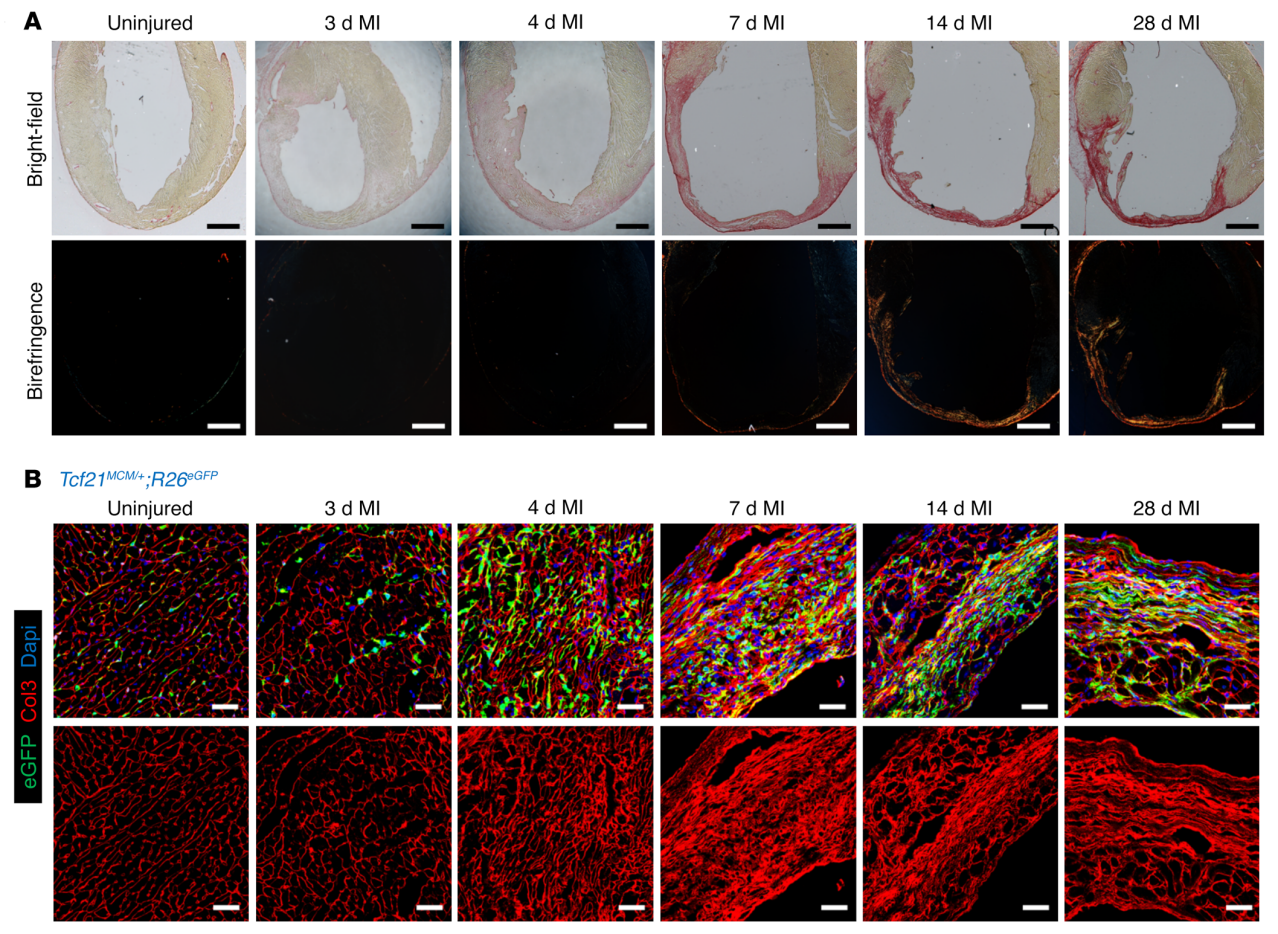
Temporal maturation of the scar in the mouse heart. (**A**) Representative Picrosirius red staining images of WT heart histological cross-sections taken in bright-field mode or under polarized light for birefringence detection at the indicated time points showing the progression of fibrosis and collagen maturation after MI injury. Scale bars: 1 mm. Representative images are shown from 3 hearts analyzed at each time point. (**B**) Representative IHC images showing morphological changes in Col3 (red) along with *Tcf21* lineage–traced (EGFP^+^) fibroblasts from the infarct region of hearts from *Tcf21^MCM/+^*;*R26^EGFP^* mice at indicated time points. Nuclei are shown with DAPI (blue). Scale bars: 20 μm. Representative images are shown from 3 hearts analyzed at each time point.

**Figure 7:**
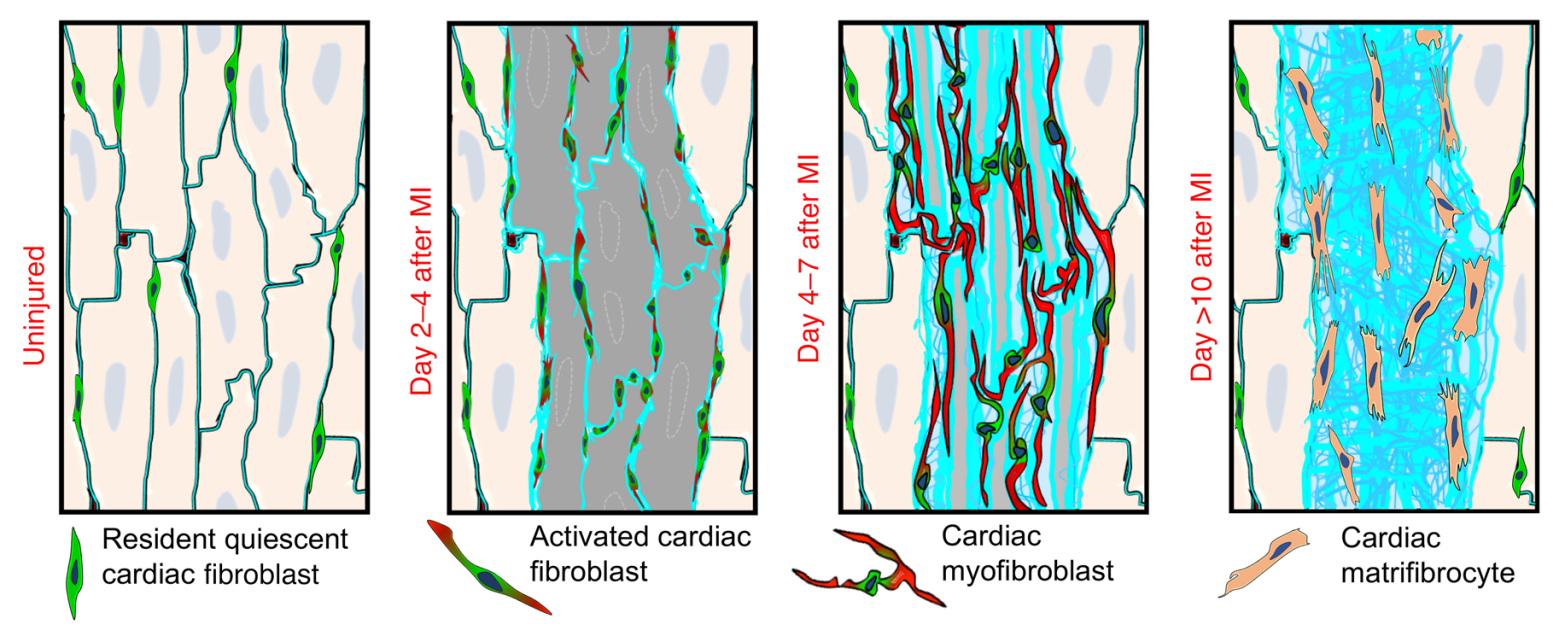
Model of MI injury–dependent cardiac fibroblast states. Model showing the different states of cardiac fibroblasts at different post-MI stages. In uninjured heart, cardiac fibroblasts reside within the interstitial space, but after MI injury, they become maximally activated by 2 to 4 days, and they elongate and begin to express αSMA. By days 4 to 7, the myofibroblast differentiated state is maximal, with high levels of αSMA protein in elongated processes within these cells. Finally, these fibroblasts stop proliferating and lose αSMA expression as they further differentiate into the matrifibrocyte by day 10 and onwards within the scar region.

Myofibroblasts within the infarcted region of the heart only express αSMA protein from approximately days 3 to 10 after MI, which was an unexpected observation, given that these lineage-traced cells persist for what appears to be the life of the scar. To better understand the dynamics of the cardiac myofibroblast after MI injury, we conducted a series of histological and mechanistic experiments. We first documented the progression of post-MI ventricular remodeling and ECM architecture to compare against fibroblast phenotyping. Picrosirius red staining of cardiac histological sections showed a progressive accumulation of collagen within the infarcted area over the first 6 days after MI and, thereafter, a progressive condensation of the collagen scar and an increase in its complexity (Figure 6A and Supplemental Figure 4A). Between 1 and 2 weeks, the greatest increase in collagen complexity was observed, which was also the time when myofibroblasts lost their αSMA expression (Figure 6A and Supplemental Figure 4A). Microscopic histologic analysis for collagen III (Col3) showed that between days 4 and 10, the collagen-rich basal lamina that previously surrounded each cardiomyocyte became progressively thicker and reorganized from circular structures into long continuous strands of collagen (Figure 6B, Supplemental Figure 4B, and Figure 7). The resident fibroblasts previously labeled with the *Tcf21^MCM/+^*;*R26^EGFP^* alleles initially localized around the basal lamina with long processes, but by day 10 and onwards, they reorganized commensurate with the loss of αSMA expression into more linear-shaped cells lacking processes (Figure 6B, Supplemental Figure 4B, and Figure 7). These observations suggest that αSMA expression might underlie a structural role within the first 7 days after MI injury by fully surrounding and connecting all the “ghost” basal lamellar regions with a continuous network of cellular αSMA filaments (Figure 7).

**Figure 8:**
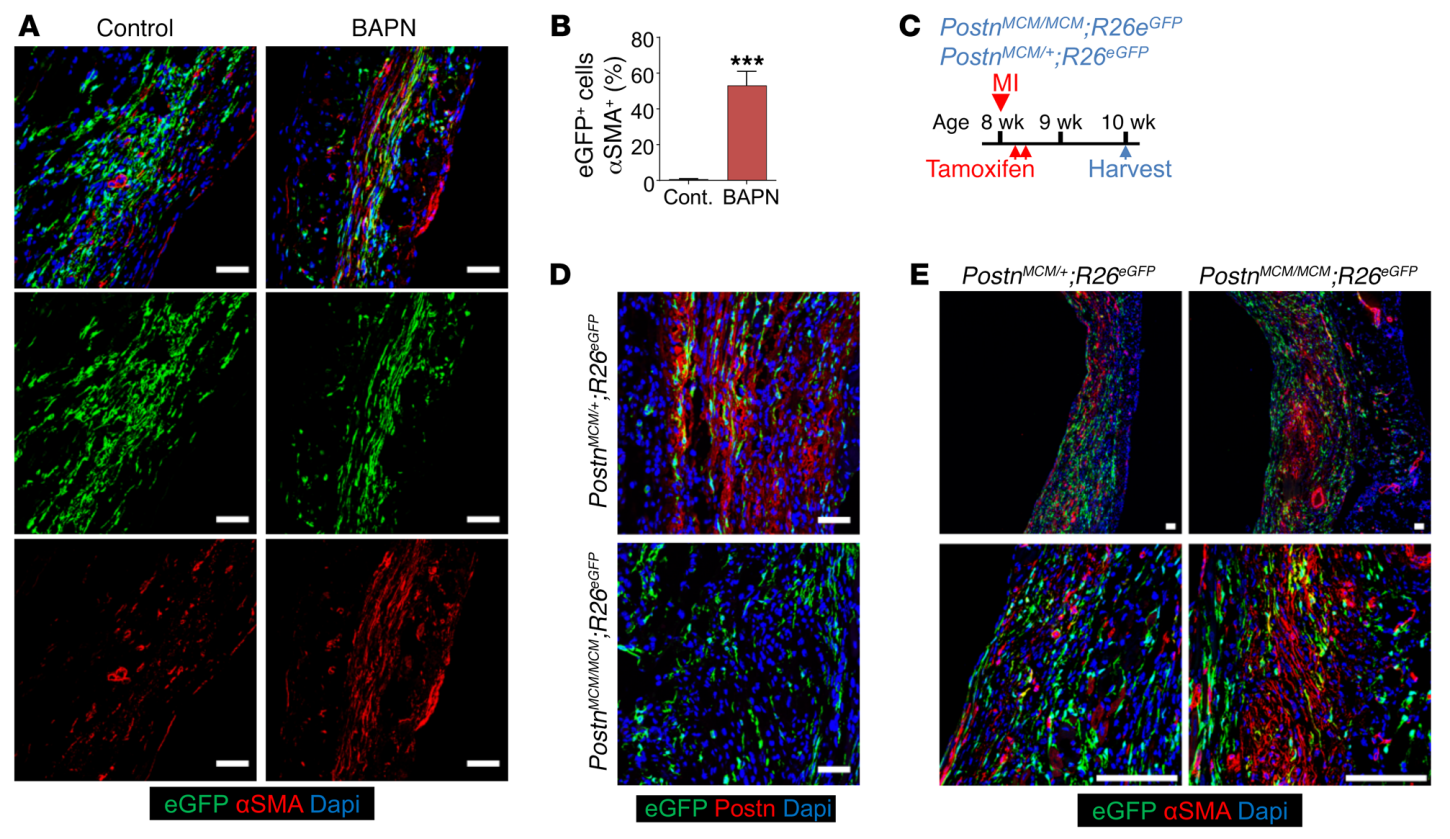
Effect of ECM maturation on αSMA expression in myofibroblasts. (**A** and **B**) Representative IHC images (**A**) and quantitation (**B**) of αSMA^+^ (red) *Tcf21* lineage–traced (EGFP^+^) fibroblasts from the infarct region of hearts from *Tcf21^MCM/+^*;*R26^EGFP^* mice treated with BAPN or PBS as a control (Cont.). Nuclei are shown with DAPI (blue). Scale bars: 20 μm. Data are shown as mean ± SD (*n* = 3). ***P < 0.0001, 2-tailed *t* test. (**C**) Experimental scheme of tamoxifen treatment of *Postn^MCM/MCM^*;*R26^EGFP^* and *Postn^MCM/+^;R26^EGFP^* mice from day 2 to day 3 after MI by daily i.p. injections. Hearts were then harvested at 2 weeks after MI. (**D**) Representative IHC images from 3 separate hearts analyzed for Postn protein expression within the infarct region of hearts from *Postn^MCM/+^*;*R26^EGFP^* mice versus *Postn^MCM/MCM^*;*R26^EGFP^* mice 2 weeks after MI. Nuclei are shown with DAPI (blue). Scale bars: 20 μm. *Postn* lineage–traced cells (EGFP^+^) cells are also shown. (**E**) Representative IHC images from 3 separate hearts analyzed showing αSMA^+^ (red) and Postn lineage–traced (EGFP^+^) fibroblasts in the infarct region of hearts from *Postn^MCM/+^*;*R26^EGFP^* mice and *Postn^MCM/MCM^*;*R26^EGFP^* mice 2 weeks after MI. Nuclei are shown with DAPI (blue). Scale bars: 50 μm.

To examine this concept of a cellular support network more directly, we used 2 different methods to destabilize maturing collagen within the infarct region, both of which resulted in prolonged αSMA expression and the traditional myofibroblast phenotype (Figure 8, A–E, and Supplemental Figure 4, C and D). The first method involved treating mice after MI with β-aminoproprionitrile (BAPN), an inhibitor of lysyl oxidase (LOX) that antagonizes collagen maturation (32, 33). This treatment maintained αSMA expression within the *Tcf21* lineage–traced fibroblasts through 14 days after MI, while αSMA expression in hearts not treated with BAPN only showed αSMA in vessels (Figure 8, A and B). We also previously showed that deletion of the *Postn* gene rendered hearts with immature scars that ruptured as well as with less crosslinked and complex collagen (34–37). Here, we generated *Postn*-null mice by crossing 2 *Postn^MCM/+^* alleles together along with the *R26^EGFP^* lineage–tracing allele. *Postn^MCM/MCM^*;*R26^EGFP^* or control *Postn^MCM+^*;*R26^EGFP^* mice were given MI at 8 weeks of age, followed by tamoxifen administration to label activated fibroblasts, after which the hearts were harvested at day 14 (Figure 8C). Importantly, *Postn^MCM/MCM^* mice showed loss of periostin protein expression in the infarct region (Figure 8D) along with reduced Col1 and Col3 within the infarct (Supplemental Figure 4D).The data again showed persistent αSMA expression in fibroblasts from *Postn*-null mice within the MI region at 14 days, while controls showed almost complete loss of αSMA expression in these fibroblasts (Figure 8E). Taken together, these results suggest that loss of the myofibroblast phenotype is associated with infarct scar maturation, such that the differentiated state of these cells with αSMA expression persists as a likely attempt to maintain ventricular wall integrity until the collagen is fully supportive.

### Gene expression defines fibroblast-differentiated states after MI.

**Figure 9:**
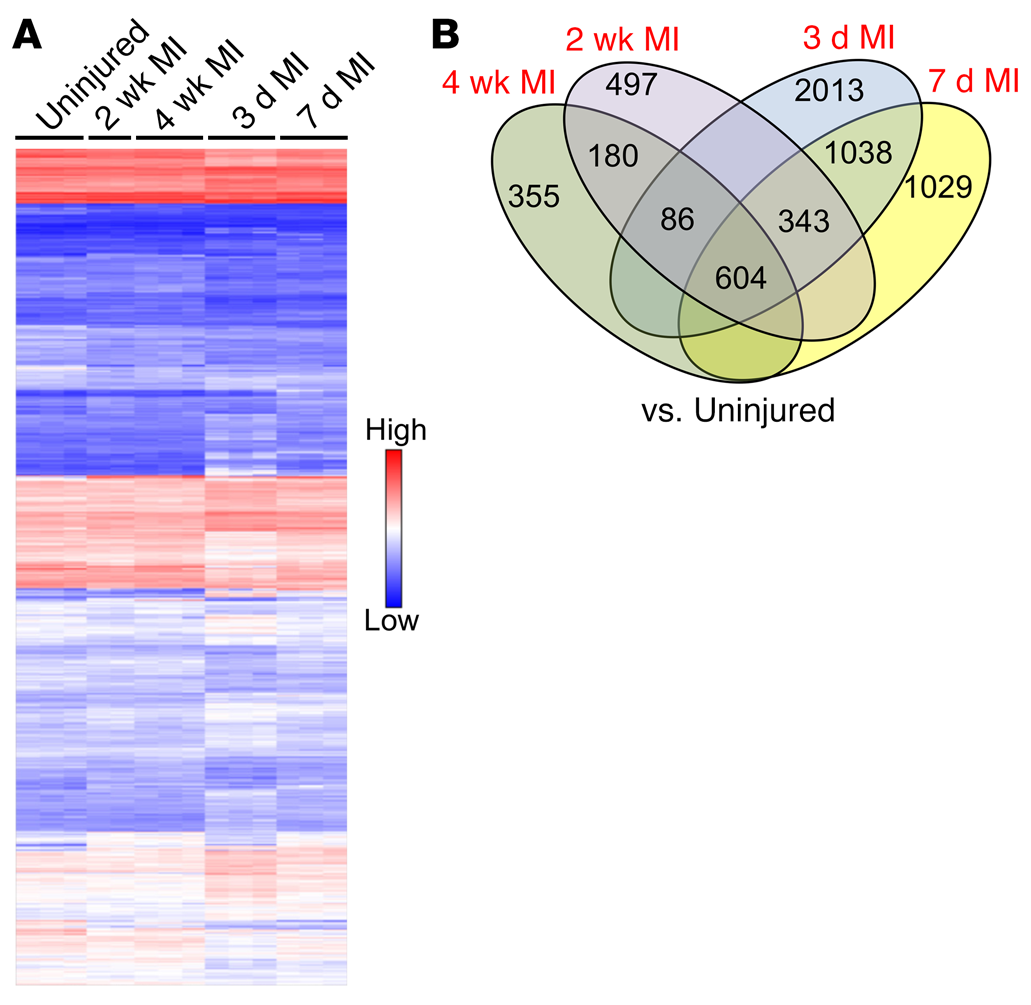
Gene-expression patterns of fibroblast stages in the MI injured heart. (**A**) Heatmap showing approximately 5,000 genes differentially expressed with cluster analysis among quiescent *Tcf21* lineage–traced (EGFP^+^) fibroblasts isolated from the uninjured LV or the MI region 3 days, 7 days, 2 weeks, and 4 weeks after injury. Individual biological replicates are shown. Individual samples were clustered and connected by Transcriptome Analysis Console based on their similarity in gene-expression patterns. (**B**) Venn diagram showing numbers and overlapping genes that were differentially expressed between *Tcf21* lineage–traced (EGFP^+^) fibroblasts isolated from the uninjured LV versus the other 4 sample groups of isolated fibroblasts from the MI region 3 days, 7 days, 2 weeks, or 4 weeks after injury.

We next performed mRNA profiling to gain insight into molecular signatures of gene expression that might underlie the phenotypic stages of fibroblasts we observed in the post-MI heart. *Tcf21^MCM+^*;*R26^EGFP^* mice were used so that EGFP^+^ fibroblasts could be directly FACS isolated for mRNA extraction without culturing. *Tcf21* lineage–traced fibroblasts were obtained from adult uninjured hearts and compared against cells isolated from the infarcted regions of hearts at 3 days, 7 days, 2 weeks, and 4 weeks, revealing some 5,000 differentially expressed genes (Figure 9A). Bioinformatic analysis showed the greatest number of gene-expression clusters at 3 days after MI injury compared with fibroblasts from uninjured hearts, followed thereafter by 7 days after MI injury, while 2 weeks and 4 weeks after MI injury showed the least number of clusters of differentially expressed genes compared with fibroblasts from the uninjured heart (Figure 9A). Indeed, Venn diagram analysis showed 2,013 uniquely differentially expressed genes between fibroblasts from the uninjured heart and 3 days after MI injury and 1,029 at day 7 after MI injury (Figure 9B). However, by 2 and 4 weeks after MI injury, there were only 497 and 355 uniquely changed genes that were significant versus those in uninjured fibroblasts, respectively (Figure 9B). Moreover, only 690 (86+604) significantly changed genes were in common between the 2- and 4-week post-MI fibroblasts compared with the 3-day post-MI activated fibroblasts (Figure 9B). These results suggest that fibroblasts from the 3-day injured MI region have the greatest differences in gene expression compared with any other time point and that by 4 weeks after MI injury, these cells are most highly related to the uninjured state, although they clearly maintain a large number of differential clusters (Figure 9, A and B). Importantly, 604 genes were always differentially expressed between fibroblasts from all 4 stages of injury with uninjured fibroblasts, showing that they are all still fundamentally the same cell lineage (Figure 9B).

**Figure 10:**
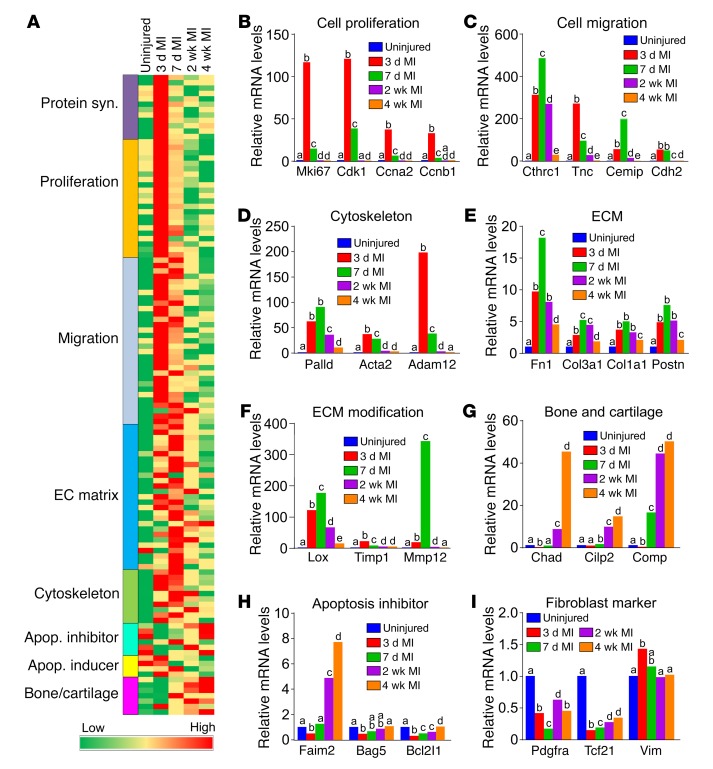
Changes in gene-expression states in the cardiac fibroblast after infarction injury. (**A**) Heatmap of selected gene-expression categories and individual genes from *Tcf21* lineage–traced fibroblasts isolated from the uninjured heart or the infarct area of hearts 3 days, 7 days, 2 weeks, and 4 weeks after MI (assayed with Affymetrix microarrays). (**B**–**I**) Bar graphs show mRNA expression levels of selected cell proliferation–related genes (**B**), cell migration–related genes (**C**), cytoskeleton-related genes (**D**), ECM protein genes (**E**), ECM modification genes (**F**), bone and cartilage–related genes (**G**), apoptosis inhibitor genes (**H**), and fibroblast marker genes (**I**) in the different sample groups. Data were normalized to quiescent *Tcf21* lineage–traced fibroblasts isolated from uninjured hearts. *n* = 3 (uninjured, 3 d MI, 7 d MI, and 4 wk MI), *n* = 2 (2 wk MI). Different letters (a, b, c, d, e) above the bars indicate significant differences (*P* < 0.05) by 1-way ANOVA and Tukey’s post hoc analysis between *Tcf21* lineage–traced fibroblasts isolated at different time points in each panel. Bars denoted by the same letter indicate lack of significant difference within that panel, while all bars that have different letters are significantly different from each other.

Genes were also clustered into key biologic features of the fibroblast, such as proliferation, cell migration, cytoskeleton, ECM and ECM modification, apoptosis inhibitor, and bone and cartilage signatures (Figure 10A). Consistent with our phenotypic data, proliferation- and migration-regulating genes were most highly induced at day 3 after MI in *Tcf21* lineage–traced fibroblasts, but were then extinguished by 2 and 4 weeks (Figure 10, B and C). Cytoskeletal- and ECM-modifying genes were also most highly induced at days 3 and 7, but then downregulated by 4 weeks after MI (Figure 10, D and F), while traditional fibroblast marker genes and ECM genes remained expressed at essentially all time points (Figure 10, E and I). However, we observed that Tcf21 and Pdgfrα were most highly expressed in quiescent fibroblasts and became downregulated with fibroblast activation (Figure 10I). Apoptosis inhibitory genes were also induced by day 7 and onwards, perhaps providing protection of these cells in the relatively hypoxic environment of the scar (Figure 10H). Finally, we also identified a number of secreted genes that were differentially expressed (Supplemental Figure 5), but across the different “fibroblasts stages,” we did not observe a change in inflammatory pathway genes or a clustering of immune response genes (data not shown).

**Figure 11:**
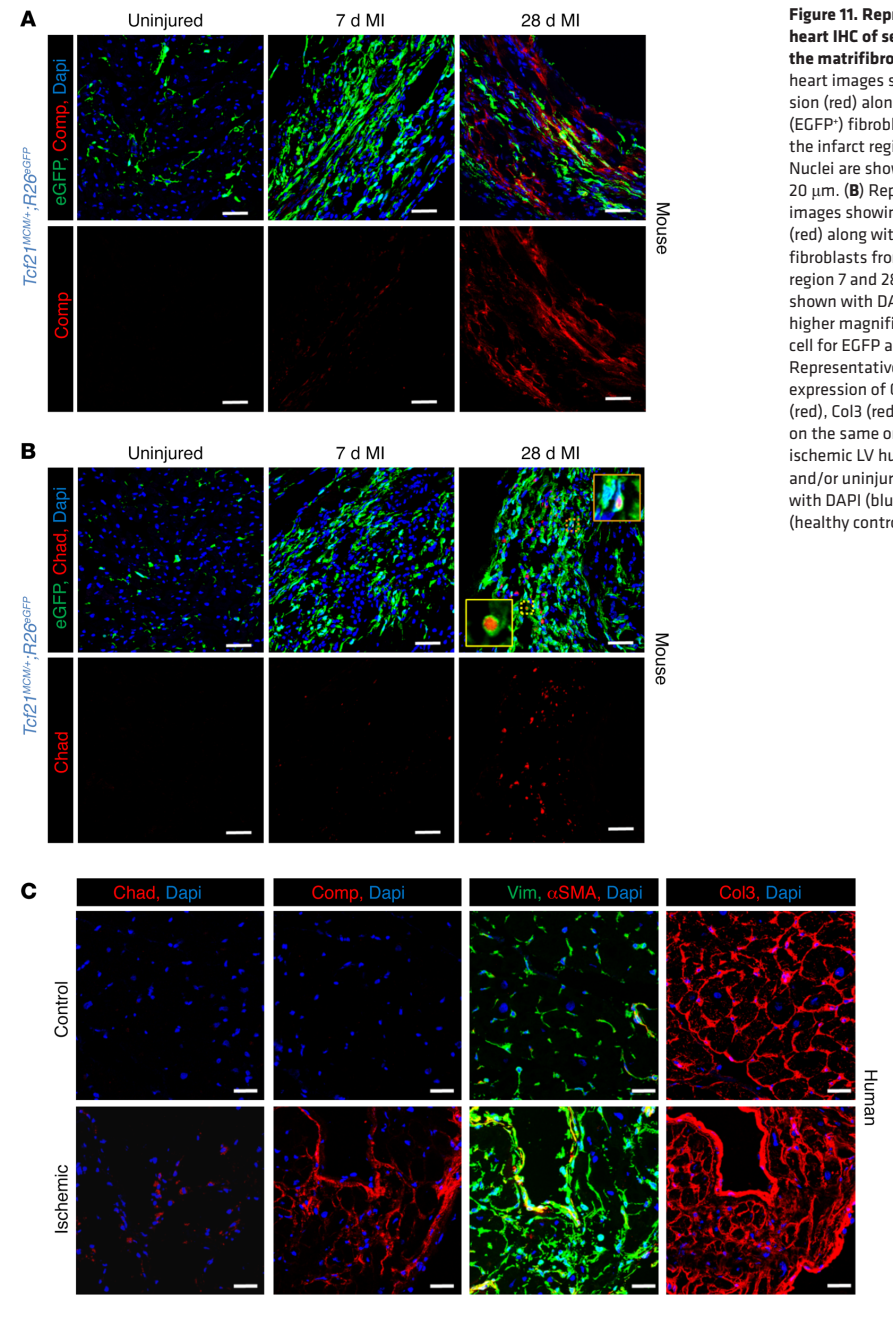
Representative mouse and human heart IHC of selected proteins underlying the matrifibrocyte. (**A**) Representative IHC heart images showing Comp protein expression (red) along with *Tcf21* lineage–traced (EGFP^+^) fibroblasts from uninjured mice or the infarct region 7 and 28 days after MI. Nuclei are shown with DAPI (blue). Scale bars: 20 μm. (**B**) Representative IHC mouse heart images showing Chad protein expression (red) along with *Tcf21* lineage–traced (EGFP^+^) fibroblasts from uninjured heart or the infarct region 7 and 28 days after MI. Nuclei are shown with DAPI (blue). The insets show higher magnification (×4) of double-positive cell for EGFP and Chad. Scale bars: 20 μm. (**C**) Representative IHC images showing protein expression of Chad (red), Comp (red), αSMA (red), Col3 (red), or vimentin (Vim, green) on the same or adjacent serial sections of ischemic LV human heart samples with scar and/or uninjured (control). Nuclei are shown with DAPI (blue). *n* = 3 (ischemic LVAD). *n* = 1 (healthy control). Scale bars: 20 μm.

While the most gene changes were observed at 3 days after MI injury versus those in fibroblasts from the uninjured heart, bioinformatic pathway ontology analysis showed a remarkable profile of up- and downregulated genes in fibroblasts isolated from the post-MI heart at 4 weeks that appeared to represent an entirely unique differentiated state (Supplemental Table 1). Most notable among these pathway gene changes was an induction of genes underlying bone, connective tissue, cartilage, and tendon development or processing (Supplemental Table 1). Selected genes from this pathway signature at 2 and 4 weeks after MI showed an induction of cartilage oligomeric matrix protein (Comp, also known as thrombospondin 5) and chondroadherin (Chad) and Cilp2 (Figure 10G). IHC staining confirmed the specific expression of Comp and Chad in the infarct region at 4 weeks after MI, with little to no protein expression observed at day 7 when myofibroblasts are present (Figure 11, A and B). Importantly, Comp and Chad expression were also identified in the fibrotic area of human heart samples from ischemic patients that had left ventricular (LV) assist devices placed, but not within unscarred or uninjured heart tissue (Figure 11C). Also of note, αSMA expression was not observed in vimentin^+^ cells (fibroblasts) within the mature scarred region of human hearts, although nearby vessels showed αSMA expression in vascular smooth muscle cells, presumably suggesting that myofibroblasts are also a transient cell type in the scar of the human heart (Figure 11C).

### Secondary cryoinjury and cell ablation within the scar exacerbates heart failure.

To study the potential function of matrifibrocytes, cryoinjury was applied to the infarct region 8 weeks after the initial surgical MI procedure as a means of killing these cells (Supplemental Figure 6A). Two weeks after cryoinjury, the number of *Acta2* lineage–traced matrifibrocytes was efficiently reduced, although the vascular cell content was also reduced (Supplemental Figure 6B). IHC showed that the ECM in the cryoinjured infarct region was mildly disrupted with reduced Col3 content and organization (Supplemental Figure 6B). Heart function was also significantly reduced after the cryoinjury procedure compared with that in sham controls, which remained the same as before cryoinjury (Supplemental Figure 6, C and D, *P* < 0.05). These data indirectly suggest that matrifibrocytes could have a functionally important role in maintaining the integrity of the mature scar.

## Discussion

Despite their importance in cardiac disease states, surprisingly few studies are present in the literature that interrogate the cellular dynamics and function of fibroblasts in the heart with MI injury. This is likely attributable to the fact that the field previously lacked rigorous genetic approaches to reliably identify fibroblasts and their various differentiated stages in the heart. Here, we used 3 different lineage-tracing Cre-expressing lines to definitively identify quiescent resident cardiac fibroblasts, newly activated cardiac fibroblasts, or myofibroblasts for in-depth analysis as to how these cells proliferate and differentiate in the heart following MI injury. We observed that fibroblasts and cardiomyocytes are equally killed within the ischemic region, but proliferating fibroblasts in the border zone at day 2 invade the necrotic area, where by days 3 to 4, they achieve maximal proliferation and coverage within the developing scar. The proliferation of activated fibroblasts or myofibroblasts remains high for another 2 to 3 days within the infarct region, but is dampened 1 day sooner within the border zone region. Unexpectedly, proliferation of activated fibroblasts and myofibroblasts is almost entirely extinguished by 7 days after MI injury, which is consistent with a very recent report (38), although dynamic remodeling of the scar area with increased collagen complexity continues through week 2. Remarkably, mechanical stress itself robustly induces the differentiation of quiescent fibroblasts into αSMA-expressing myofibroblasts (39). However, the stiffness and mechanical tension properties of the infarct scar gradually increase after MI (40), despite the fact that the myofibroblast differentiated state is rapidly lost by days 7 to 10 in this region.

Another interesting observation is that myofibroblasts with abundant αSMA expression proliferate (days 3 to 6), which is consistent with a previous report on the infarcted heart (21). However, previous studies suggested that myofibroblasts within the stabilized infarct scar were cleared by apoptosis (41, 42), although we observed that lineage-traced fibroblasts (*Tcf21*-MCM), activated fibroblasts (*Postn*-MCM), and myofibroblasts (*Acta2*-CreERT2) persist within the scar long term and are highly resistant to apoptosis. The discordance is likely because the approaches by Takemura et al. and Zhao et al. (41, 42) were not based on lineage tracing and because loss of αSMA expression might have been incorrectly interpreted as cell loss. Finally, we observed that fibroblast proliferation resulted in an approximate 3.5-fold total expansion of these cells within the infarction area compared with the uninjured heart and that this enhanced cell content was maintained long term within the scar without cell turnover.

Another interesting observation of the current study is that αSMA expression is limited to the first 7 to 10 days of MI injury within myofibroblasts, indicating that this differentiated state is rather transient in the scar area. Myofibroblasts with αSMA expression have been shown to underlie tissue regeneration in the lung and skin, and αSMA expression decreases as the regeneration process is completed (9, 43, 44). We have previously shown that deletion of activated fibroblasts from the infarcted mouse heart using *Postn^MCM/+^*;*R26DTA* alleles resulted in much greater lethality due to ventricular wall rupture, indicating that activated fibroblasts and myofibroblasts are required for acute ventricular wall healing (23). Even mice with deletion of the *Postn* gene, which is needed for collagen maturation (34), exhibit a heart significantly more susceptible to ventricular wall rupture in the first week after MI injury (37, 45). Thus, activated fibroblasts and myofibroblasts are critical players in cardiac wound healing after MI injury.

We previously showed that myofibroblasts in the heart can dedifferentiate and revert back to an “uninjured” state after a cardiac stress response resolves, such as with Ang II/PE agonist infusion and then its removal (23). A similar response is also observed in regenerative tissues such as lung, in which an injury is fully resolved and fibrosis typically reverts back to preinjury status (9). In comparison, MI injury requires the persistence of a stable star because the adult mammalian heart is not significantly regenerative. This stable scar environment houses the more highly differentiated fibroblast stage referred to here as the matrifibrocyte (Figure 7). These cells share some gene signatures with chondrocytes and osteoblasts, which are adapted to highly collagenous environments and for building and maintaining cartilage and bone ECM (46–48). However, extensive biologic pathway analysis of the 5,000 differentially expressed and clustered genes suggests that these cells are entirely unique and are not chondrocytes, osteoblasts, osteoclasts, tendon cells, or myofibroblasts, although they are most closely related to quiescent fibroblasts (Supplemental Table 1, and see Figure 9, A and B). The expression of ECM and ECM-modifying genes, including some genes typically observed in tendon, bone, and cartilage, suggests that matrifibrocytes within the scar are more highly specialized for this environment. Matrifibrocytes within the scar also resist new rounds of proliferation when challenged with Ang II/PE, yet fibroblasts in the uninjured regions of the heart show abundant proliferation.

To fully explore the functional role of these cells, we first attempted to selectively kill them using a *Rosa26-loxP-DTR-loxP* approach in conjunction with the *Postn^MCM^* allele. Using these alleles, expression of the human diphtheria toxin receptor was induced on all activated fibroblasts, which we later attempted to kill coincident with infusion of diphtheria toxin. Importantly the mouse is normally resistant to diphtheria toxin unless the human receptor is expressed (49). However, for unknown technical reasons, this system was not effective in killing matrifibrocytes in the scar (data not shown); hence, the less specific cryoinfarction model was necessary. This later model did effectively kill matrifibrocytes in the scar and was suggestive of a potential important functional role for these cells in maintaining the integrity of the mature scar, although vascular cells in the scar were also killed by this procedure and could have also contributed to the reductions in scar integrity. Finally, the human scar also appears to contain matrifibrocytes based on loss of αSMA expression in vimentin^+^ cells and attainment of Chad and Comp expression, suggesting that these cells warrant further mechanistic investigation as another potential strategy for positively affecting post-MI scar formation and long-term scar remodeling with heart failure.

## Methods

### Human samples.

Human myocardial LV free wall tissue was obtained from patients of ischemic etiology (*n* = 3) during LV assist device implantation. Nonfailing LV free wall tissue was obtained from donors deemed unsuitable for transplant with no cardiac dysfunction. Tissue was freshly obtained and embedded in OCT compound and frozen for cryosectioning.

### Mice.

Mouse lines used in this study were as follows: *Postn*-MerCreMer (23), *Tcf21*-MerCreMer (26), *Acta2*-CreERT2 (29), *Postn-*LacZ (Jackson Laboratories, stock no. 024186), and FVB.Cg-*Gt(ROSA)26Sortm1(CAG-EGFP)Glh*/J (50). *Postn*-MerCreMer mice, *Tcf21*-MerCreMer mice, and *Acta2*-CreERT2 mice were crossbred with FVB.Cg-*Gt(ROSA)26Sortm1(CAG-EGFP)Glh*/J mice to obtain *Tcf21^MCM/+^*;*R26^EGFP^* mice, *Postn^MCM/+^*;*R26^EGFP^* mice, and *Acta2^CreERT2^*;*R26^EGFP^* mice, respectively. *Tcf21^MCM/+^*;*R26^EGFP^* mice were crossbred with *Postn-*LacZ mice to obtain *Tcf21^MCM/+^*;*R26^EGFP^*;*Postn^LacZ/+^* mice.

### Antibodies and biologics.

Antibodies against EGFP (catalog ab13970 and ab290), β-galactosidase (catalog ab9361), Col1 (catalog ab34710), vimentin (catalog ab45939), and Col3 (catalog ab7778) were purchased from Abcam. Anti-αSMA antibody (catalog A2547), anti-Chad antibody (catalog HPA018241), Ang II (catalog A9525), and PE hydrochloride (catalog P6126) were purchased from Sigma-Aldrich. Anti-Ki67 antibody (catalog 9129S) was purchased from Cell Signaling Technology. Anti-periostin antibody (catalog NBP1-30042) was purchased from Novus Biologicals. Anti-CD45 antibody (catalog 14-0451-82) was purchased from eBioscience. Anti-CD31 antibody (catalog 553370) was purchased from BD Bioscience. Anti-Comp antibody (catalog 13641-1-ap) was purchase from ProteinTech. Goat anti-mouse IgG2a Alexa Fluor 568 (catalog A-21134), goat anti-chicken Alexa Fluor 488 (catalog A-11039), goat anti-chicken Alexa Fluor 568 (catalog A-11041), goat anti-rabbit Alexa Fluor 488 (catalog A-11008), and goat anti-rabbit Alexa Fluor 568 (catalog A-11036) secondary antibodies were purchased from Life Technologies. TGF-β (catalog 101-b1-010) was purchased from R&D Systems. EdU (catalog sc-284628A) was purchased from Santa Cruz Biotechnology Inc. Collagenase D (catalog 11088866001) and Dispase II (catalog 10165859001) were purchased from Roche Diagnostics.

### Animal procedures.

To induce activity of the MerCreMer or the CreERT2 protein, mice were fed a diet containing 400 mg/kg tamoxifen citrate (Envigo, TD.55125) or were treated with tamoxifen (MilliporeSigma, T5648) dissolved in corn oil through i.p. injections at a dosage of 75 mg/kg body weight/d. The duration of treatment is indicated within Figure 1, A and F; Figure 3, A and C; Figure 4B; Figure 5A; and Figure 8C. MI was induced in mice via permanent surgical ligation of the left coronary artery (51). Briefly, mice were anesthetized using isoflurane and a left lateral thoracotomy was performed. The left coronary artery was identified and ligated just below the left atrium. For Ang II/PE treatment, micro-osmotic pumps (Azlet, model 1002) were inserted s.c., delivering a combination of 1.5 μg/g/d Ang II (MilliporeSigma, A9525) and 50 μg/g/d PE hydrochloride (MilliporeSigma, P6126). Cryoinjury was induced in mice via applying a liquid nitrogen–cooled metal probe (3 mm × 3 mm) to the surgically exposed prior infarct region of the heart for 3 seconds. For LOX inhibition studies, mice were treated with BAPN through i.p. injection at a dosage of 500 mg/kg body weight/d from 1 day before MI to day 14 after MI. Mice treated with PBS were used as vehicle control. Echocardiography was performed in M-mode using a Hewlett Packard SONOS 5500 instrument equipped with a 15 MHz transducer as described previously (52). For pain management related to surgical procedures, mice were given a single postoperative dosage of buprenorphrine-sustained release formula (3.25 mg/kg body weight) by s.c. injection at 0.05 to 0.1 mg/kg.

### EdU in vivo fibroblast proliferation assay.

Mice were treated with EdU at a dosage of 50 mg/kg body weight through i.p. injections. Four hours after EdU injections, mice were sacrificed and heart samples were collected. EdU detection was carried out after IHC staining using the Click-iT Plus Alexa Fluor 647 Picolyl Azide Toolkit (C10643) from Thermo Fisher Scientific.

### FACS.

Heart tissue was digested as previously described, with modifications (53). Briefly, heart tissue was minced and digested in DMEM containing 0.75 U/ml collagenase D (Roche, 11088866001), 1.0 U/ml Dispase II (Roche, 10165859001), and 1 mM CaCl_2_ at 37°C for 40 minutes. The slurry was then passed through a 100 μm cell strainer and then a 40 μm cell strainer. Cells were collected by centrifugation at 350 *g* for 10 minutes. The cell pellet was then resuspended in PBS. *Tcf21* lineage–traced EGFP^+^ cardiac fibroblasts were sorted on FACSaria II (BD Biosciences). Gates were made based on WT EGFP^–^ control. Sorted EGFP^+^ cardiac fibroblasts were used for RNA analysis. For FACS involving αSMA and EdU staining, heart tissue was removed from mice 4 hours after EdU injection and digested and sorted as described above. Sorted EGFP^+^ cardiac fibroblasts were fixed in 100% ice-cold methanol for 30 minutes, rinsed in PBS, and blocked in PBS with 1% BSA for 20 minutes at room temperature. EdU staining was performed using Click-iT Plus Alexa Fluor 647 Picolyl Azide Toolkit (C10643) from Thermo Fisher Scientific, which was followed by incubation with anti-αSMA eFluor 570 (eBioscience 41-9760-82, 1:50) for 1 hour at room temperature. Cells were then rinsed in PBS and analyzed on FACSCanto (BD Biosciences). Undifferentiated NIH 3T3 cells and *Tcf21* lineage–traced EGFP^+^ cardiac fibroblasts sorted from uninjured hearts were processed at the same time and were used as negative control for gating.

### Cell culture.

FACS-sorted *Tcf21* lineage–traced fibroblasts were resuspended in growth medium composed of DMEM with 10% bovine growth serum and 1% of an antibiotic mixture containing 10,000 U/ml penicillin, 10 mg/ml streptomycin, and 25 μg/ml amphotericin B. Resuspended fibroblasts were seeded on 8-chamber slides at a density of 5 × 10^3^ cells/well. Cells were grown at 37°C with 5% CO_2_. For EdU-based proliferation assay, cells were cultured in the same growth medium supplemented with 10 μM EdU for 6 hours and with or without TGF-β stimulation (10 ng/ml) starting at 60 hours after cell isolation. For induction of myofibroblast differentiation, *Tcf21* lineage–traced fibroblasts were cultured in the same growth medium supplement with TGF-β (10 ng/ml) for 48 hours.

### Affymetrix microarray and bioinformatics.

Total RNA was extracted from 50,000 FACS-sorted *Tcf21* lineage–traced fibroblasts using the miRNeasy Micro Kit (217084, QIAGEN). Microarray analysis was carried out via the Gene Expression Core Facility (CCHMC) using the Affymetrix Clariom S platform. Differential gene expression between samples was determined via bioinformatics analyses of resultant data (CEL files) using the Transcriptome Analysis Console (Applied Biosystems; ver. 4.0.0.25), the Clariom_S_Mouse TAC Configuration file (ver. 1), and the iPathwayGuide (Advaita Bioinformatics). All original microarray data were deposited in the NCBI’s Gene Expression Omnibus database (GEO GSE111059).

### Immunocytochemical staining.

FACS-sorted *Tcf21* lineage–traced fibroblasts grown on multiple-chamber slides were fixed in 4% PFA for 10 minutes, rinsed 3 times in TBS with 0.1% Triton X-100, incubated in blocking buffer (TBS, 0.1% Triton X-100, and 3% BSA), and then incubated with primary antibodies diluted in blocking buffer at 4°C overnight. Cells were then rinsed in TBS with 0.1% Triton X-100 3 times and stained with corresponding secondary antibodies diluted in blocking buffer for 1 hour at room temperature. Stained slides were then rinsed and mounted in a mounting medium containing DAPI (Vector Laboratories). Images were taken using an inverted Nikon A1R confocal microscope using NIS Elements AR 4.13 software. Primary antibodies and dilutions used in immunocytochemical staining (ICC) included anti-EGFP antibody (Abcam ab13970, 1:200) and anti-αSMA antibody (MilliporeSigma A2547, 1:200). Secondary antibodies and dilutions used in ICC included goat anti-mouse IgG2a Alexa Fluor 568 (Life Technologies A-21134, 1:500) and goat anti-chicken Alexa Fluor 488 (Life Technologies A-11039, 1:500).

### IHC staining.

Mouse heart samples were fixed in 4% paraformaldehyde for 3.5 hours, rinsed in PBS for 30 minutes, and immersed in PBS containing 30% sucrose overnight at 4°C before being embedded in OCT (Tissue-Tek) for cryosectioning. Human heart samples were from LV free wall plugs excised during LV assist device implantation or at the time of cardiac transplantation. Excised tissue was immediately embedded in OCT and frozen for cryosectioning. Sections of fresh-frozen tissue were fixed in 4% paraformaldehyde for 5 minutes at room temperature before being processed for staining. Sections (7 μm thick) were incubated in blocking buffer containing TBS, 5% goat serum, and 0.2% Triton X-100 for 2 hours. For IHC employing anti-αSMA antibody (MilliporeSigma, A2547, 1:200), anti-periostin antibody (Novus, NBP1-300421, 1:200), anti-vimentin (Abcam, ab45939, 1:200), or anti-Ki67 antibody (Cell Signaling Technology, 9129S, 1:200) antigen retrieval was performed by heating sections in citrate buffer (pH 6.0) for 20 minutes before blocking. Blocked sections were incubated in primary antibodies diluted in blocking buffer overnight at 4°C, rinsed 3 times in TBS containing 0.2% Triton X-100, and then incubated in appropriate fluorophore-conjugated secondary antibodies diluted in blocking buffer for 1 hour at room temperature. Sections were then rinsed 3 times in TBS containing 0.2% Triton X-100 and mounted in a mounting medium containing DAPI. Images were captured using an inverted Nikon A1R confocal microscope using NIS Elements AR 4.13 software. Additional primary antibodies and dilutions used in IHC included the following: anti-EGFP (Abcam, ab290, 1:200), anti–β-galactosidase (Abcam, ab9361, 1:200), anti-Col1 (Abcam, ab34710, 1:200), anti-Col3 (Abcam, ab7778, 1:200), anti-CD45 (eBioscience, 14-0451-82, 1:50), anti-CD31 (BD Bioscience, 553370, 1:100), anti-Chad antibody (MilliporeSigma, HPA018241, 1:100), and anti-Comp antibody (ProteinTech, 13641-1-ap, 1:100). Secondary antibodies were used at 1:500 dilution in IHC. These included goat anti-mouse IgG2a Alexa Fluor 568 (Life Technologies, A-21134), goat anti-chicken Alexa Fluor 488 (Life Technologies, A-11039), goat anti-chicken Alexa Fluor 568 (Life Technologies, A-11041), goat anti-rabbit Alexa Fluor 488 (Life Technologies, A-11008), and goat anti-rabbit Alexa Fluor 568 (Life Technologies, A-11036).

### Identification and quantification of cardiac fibroblasts in IHC staining.

Longitudinal sections containing the core infarct regions were used for IHC staining and quantification analysis. EGFP^+^ cells with nuclei identified by DAPI staining were counted as lineage-traced cardiac fibroblasts and were included in the analysis. For each heart sample, the total number of cardiac fibroblasts on 4 randomly selected microscopic fields of each of 3 stained sections at various depths representing the entire infarct region was counted (4 fields per section, 3 sections per heart). The data from 3 independent sections for each biological sample were averaged for comparison. Mice were randomly assigned to experimental groups. Experiments were performed in a blinded manner where possible.

### Picrosirius red staining.

Picrosirius red staining was carried out as previously described (54). Briefly, longitudinal heart sections were collected from frozen samples and incubated in Bouin’s fixative (Electron Microscopy Sciences, 26367-01) for 1 hour at 55°C and then stained with Picrosirius red (Electron Microscopy Science, 26357-02) for 1 hour at room temperature. Sections were subsequently dehydrated and cleared with xylene. Pictures were captured in bright-field mode or under polarized light to capture the birefringence of collagen fibers using an Olympus light microscope.

### Statistics.

All data are expressed as mean ± SEM unless otherwise stated. Data were analyzed using GraphPad Prism 7 (GraphPad Software, Inc.). One-way ANOVA with post hoc Tukey’s test was used to determine the significance of difference when more than 2 groups were compared. Two-tailed *t* test was used to determine significance of differences among means when only 2 groups were compared. *P* < 0.05 was considered significant.

### Study approval.

All experiments involving mice were approved by the IACUC at CCHMC (approval number IACUC 2016-0069). Experimentation with human tissue was performed in accordance with NIH and HIPAA guidelines under an approved Institutional Review Board protocol (2013-1386 of CCHMC to Burns Blaxall). Patients gave informed consent.

## Author contributions

JDM and XF conceived and designed the study. Experiments were performed by XF, OK, HK, RJV, JGB, BDM, and MAS. VP performed bioinformatics analysis. BCB and IVA provided human heart samples. JDM and XF wrote the manuscript with input from all authors.

## Supplementary Material

Supplemental data

## Version 1 04/16/2018

Electronic publication

## Version 2 05/01/2018

Print issue publication
