# Supplementary material for: Specialized fibroblast differentiated states underlie scar formation in the infarcted mouse heart
Source: J Clin Invest. 2018 Apr 16;128(5):2127–43. doi: 10.1172/JCI98215 (PMC5957472; doi:10.1172/JCI98215)
Supplement: Supplemental data [file jci-128-98215-s001.pdf]

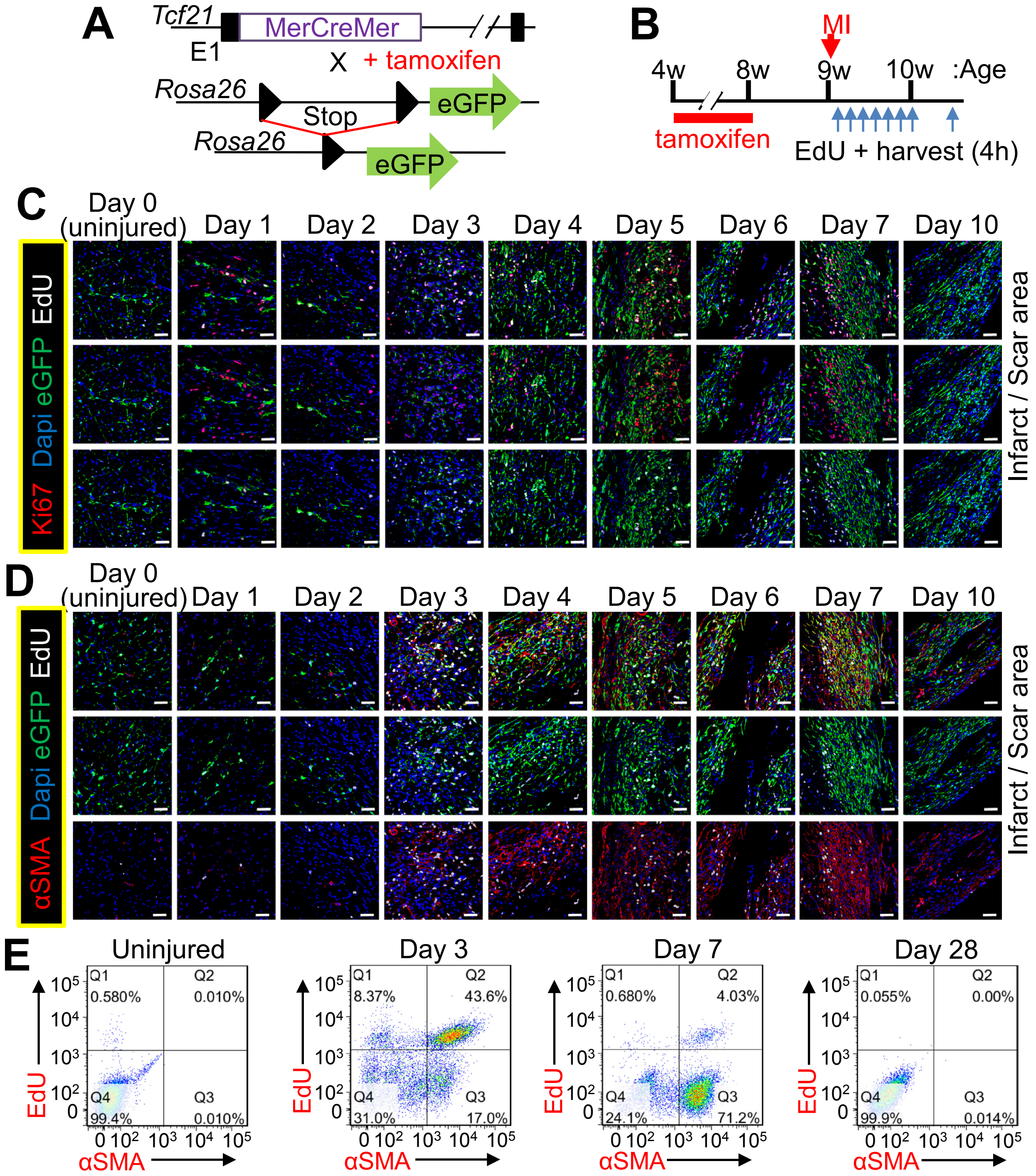

Supplemental Figure 1. IHC heart staining showing *Tcf21* lineage-traced fibroblast proliferation during the first 10 days post-MI. (**A**) Schematic of the *Tcf21* locus containing a tamoxifen-regulated MerCreMer cDNA cassette inserted into exon 1 (E1), which was crossed with $R26^{eGFP}$ reporter mice containing a loxP site-flanked stop cassette upstream of eGFP to allow for Cre-dependent lineage tracing. (**B**) Experimental scheme whereby $Tcf21^{MCM/+}$;$R26^{eGFP}$ mice were given tamoxifen for 4 weeks and rested for 1 week before MI surgery. Mice were treated with a single EdU injection at the indicated time points post-MI and hearts were harvested 4 hours after EdU injections for IHC analysis. (**C**) Representative IHC images showing EdU^+ (white) and $Ki67^+$ (red) versus *Tcf21* lineage-traced ($eGFP^+$) fibroblasts in uninjured hearts and after infarction injury for the indicated days. EdU staining is shown in white. Nuclei are shown with Dapi (blue). Scale bars = 20 µm. Days 0, 2, 3, 4, and 7 images are also shown in Figure 1E and reproduced here for completeness. (**D**) Representative IHC images showing $αSMA^+$ (red) and EdU^+ (white) versus *Tcf21* lineage-traced ($eGFP^+$) fibroblasts within the infarct region of hearts at day 1 to day 10 post-MI. EdU staining is shown in white. Nuclei are shown with Dapi (blue). Scale bars = 20 µm. Days 0, 2, 3, 4, and 10 images are also shown in Figure 2C and reproduced here for completeness. (**E**) Representative FACS analysis of EdU^+ and $αSMA^+$ *Tcf21* lineage-traced fibroblasts in uninjured hearts and within the infarct region of hearts at day 3, day 7, and day 28 post-MI. n = 3.

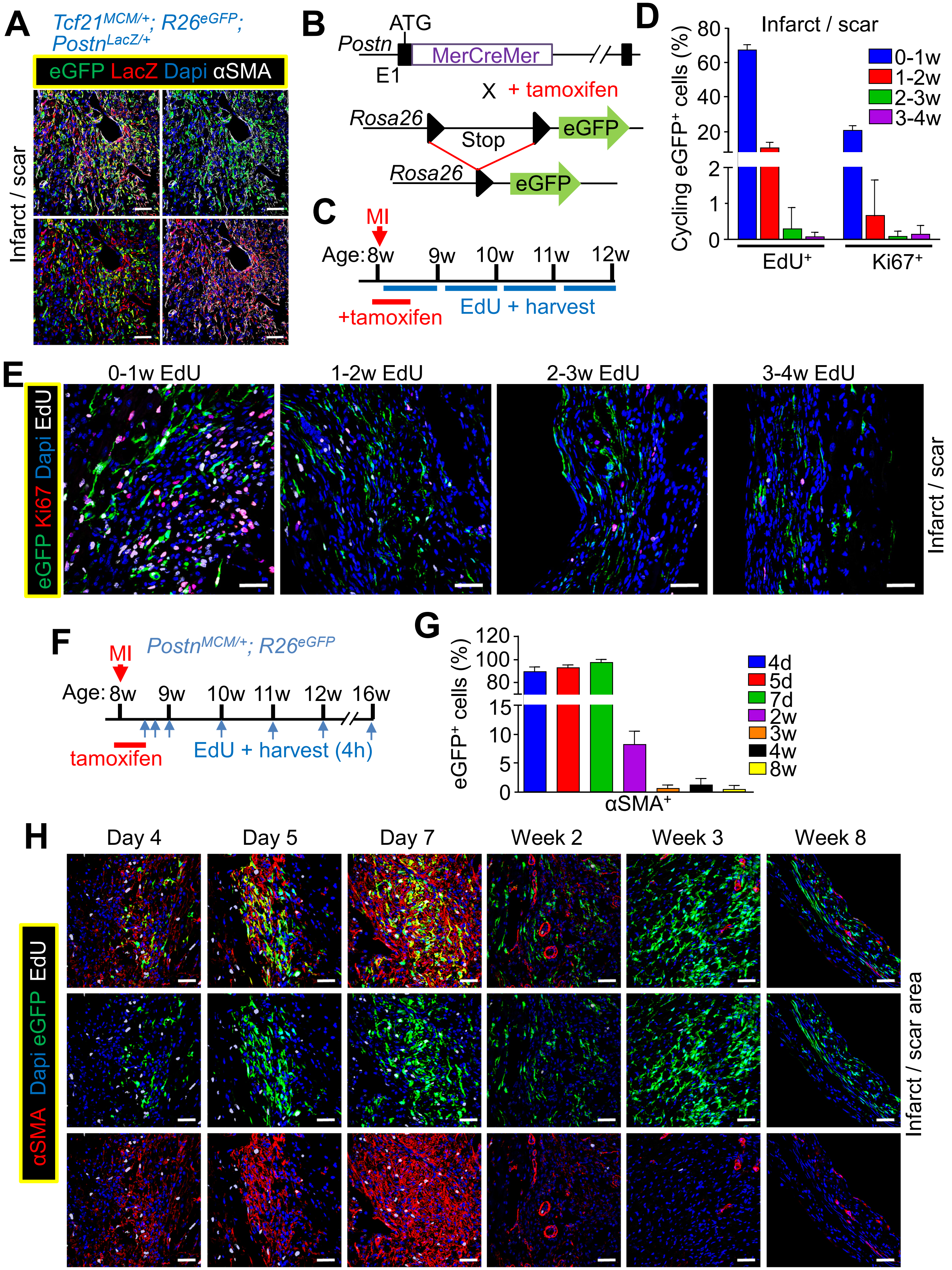

A
Tcf21MCM/+; R26eGFP; PostnLacZ/+
eGFP LacZ Dapi αSMA
Infarct / scar
B
ATG
Postn
E1
MerCreMer
X + tamoxifen
Rosa26
Stop
eGFP
Rosa26
eGFP
C
MI
Age: 8w 9w 10w 11w 12w
+tamoxifen
EdU + harvest
D
Infarct / scar
Cycling eGFP+ cells (%)
0-1w
1-2w
2-3w
3-4w
EdU+
Ki67+
E
0-1w EdU
1-2w EdU
2-3w EdU
3-4w EdU
eGFP Ki67 Dapi EdU
Infarct / scar
F
MI
PostnMCM/+; R26eGFP
Age: 8w 9w 10w 11w 12w 16w
tamoxifen
EdU + harvest (4h)
G
eGFP+ cells (%)
αSMA+
4d
5d
7d
2w
3w
4w
8w
H
Day 4
Day 5
Day 7
Week 2
Week 3
Week 8
αSMA Dapi eGFP EdU
Infarct / scar area

Supplemental Figure 2. *Postn* lineage-traced fibroblast proliferation and αSMA myofibroblast differentiation after MI. (**A**) Representative IHC heart images showing $LacZ^+$ (red) for *Postn* locus expression to compare against *Tcf21* lineage-traced ($eGFP^+$) fibroblasts within the infarct area of hearts from $TCF21^{MCM/+}$;$R26^{eGFP}$;$Postn^{LacZ/+}$ mice at day 4 post-MI. Nuclei are shown in blue (Dapi). (**B**) Schematic of the *Postn* locus with a tamoxifen regulated MerCreMer cDNA cassette inserted into exon 1 (E1), which was crossed with $R26^{eGFP}$ reporter mice containing a loxP site-flanked stop cassette upstream of eGFP to allow Cre-dependent lineage tracing. (**C**) Experimental scheme of tamoxifen treatment to $Postn^{MCM/+}$;$R26^{eGFP}$ mice from day -1 to day 3 post-MI through daily IP injections, along with 7 days of EdU injections during the indicated time periods post-MI. Hearts were harvested 4h after the last EdU injection for IHC analysis. (**D** and **E**) Quantification (**D**) and representative IHC images (**E**) of EdU^+ (white) and $Ki67^+$ (red) *Postn* lineage-traced ($eGFP^+$) fibroblasts within the MI region after 7 daily EdU injections during the indicated time periods post-MI. Dapi shows nuclei (blue). (**F**) Experimental scheme of tamoxifen treatment to $Postn^{MCM/+}$;$R26^{eGFP}$ mice from day 0 to day 3 post-MI and the single EdU injection times post-MI. Hearts were harvested 4h after EdU injections for IHC analysis. (**G** and **H**) Quantification (**G**) and representative IHC images (H) of EdU^+ (white) and $αSMA^+$ (red) versus *Postn* lineage-traced ($eGFP^+$) fibroblasts in the MI area at the indicated time points post-MI. Dapi shows nuclei (blue). (**D** and **G**) Data are mean ± SD (n = 3). (**A, E** and **H**) Scale bars = 20 μm.

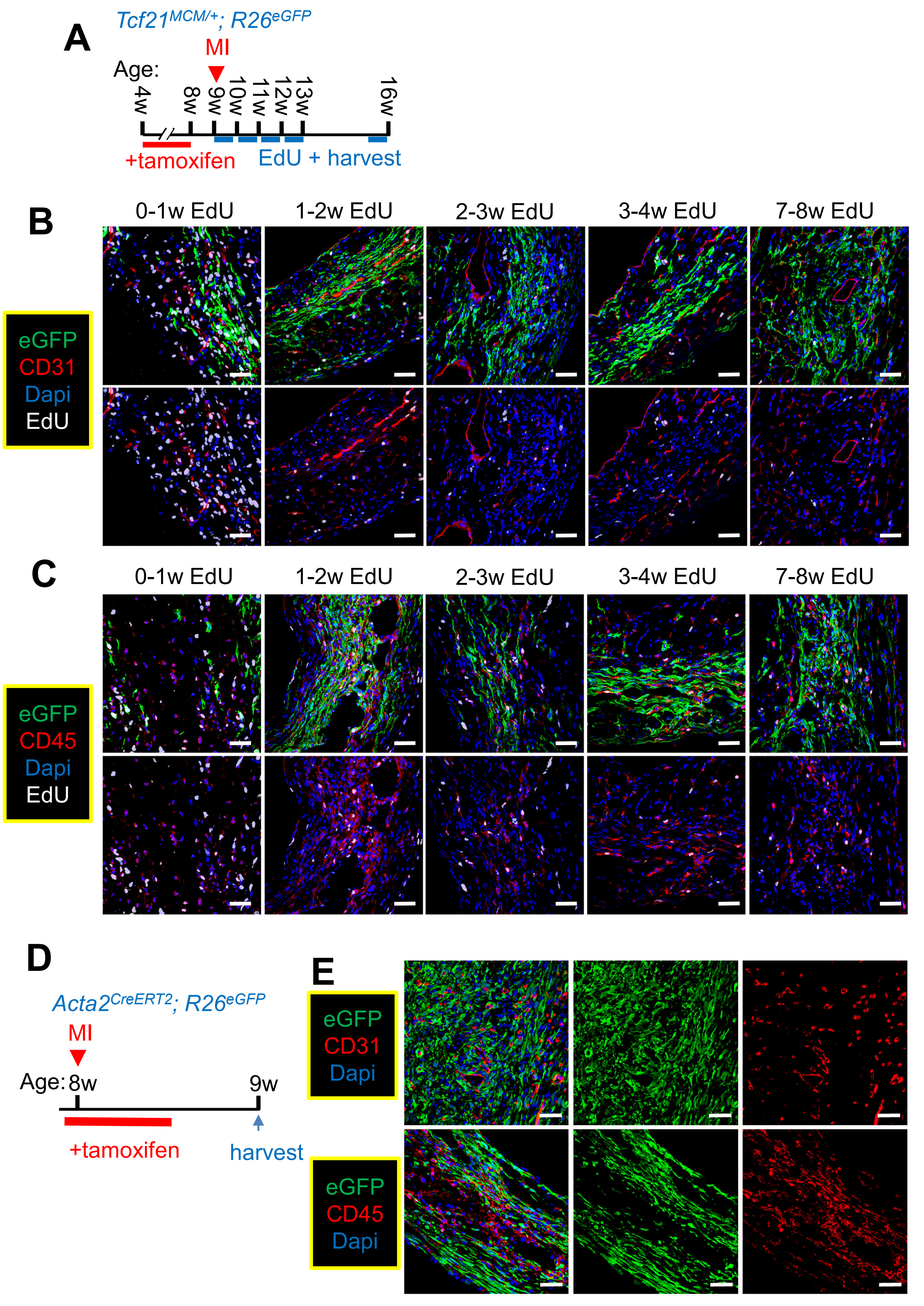
A
Tcf21MCM/+; R26eGFP
MI
Age:
4w
8w
9w
10w
11w
12w
13w
16w
+tamoxifen
EdU + harvest
B
0-1w EdU
1-2w EdU
2-3w EdU
3-4w EdU
7-8w EdU
eGFP
CD31
Dapi
EdU
C
0-1w EdU
1-2w EdU
2-3w EdU
3-4w EdU
7-8w EdU
eGFP
CD45
Dapi
EdU
D
Acta2CreERT2; R26eGFP
MI
Age: 8w
9w
+tamoxifen
harvest
E
eGFP
CD31
Dapi
eGFP
CD45
Dapi

Supplemental Figure 3. Lack of differentiation of cardiac fibroblasts to endothelial cells or leukocytes after MI. (**A**) Experimental scheme whereby *Tcf21*$^{MCM/+}$;*R26*^{eGFP} mice were given tamoxifen for 4 weeks and rested for 1 week before MI surgery. Mice were treated with 7 daily EdU injections during the indicated time periods post-MI and hearts were then harvested 4h after the last EdU injection for IHC analysis. (**B**) IHC heart images showing EdU labeled (white) CD31^{+} endothelial cells (red) versus *Tcf21* lineage-traced (eGFP^{+}) fibroblasts before MI and within the infarct region 4 and 10 days post-MI. Dapi shows nuclei (blue). (**C**) IHC heart images showing EdU labeled (white) CD45^{+} leukocytes (red) and *Tcf21* lineage-traced (eGFP^{+}) fibroblasts before MI, and within the infarct region 4 and 10 days post-MI. Dapi shows nuclei (blue). (**D**) Experimental scheme whereby *Acta2*$^{CreERT2/}$;*R26*^{eGFP} mice were given tamoxifen through daily IP injections from day 0 to day 4 after MI. Hearts were harvested at day 7 post-MI. (**E**) IHC heart images showing CD31^{+} endothelial cells (red, upper panels), CD45^{+} endothelial cells (red, lower panels), and αSMA lineage-traced eGFP myofibroblasts within the MI region 7 days post-MI. Dapi shows nuclei (blue). Scale bars = 20 μm.

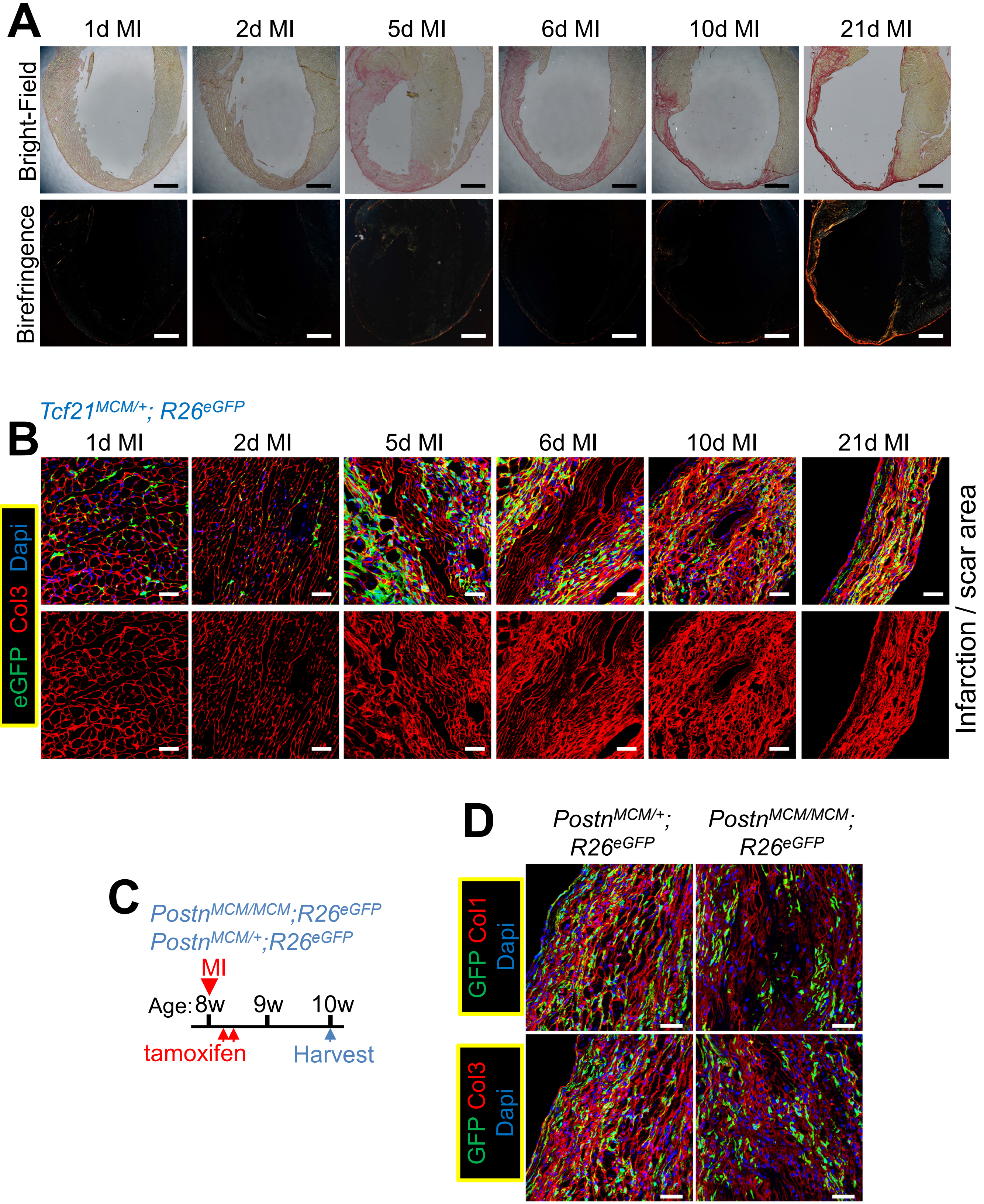
A
1d MI
2d MI
5d MI
6d MI
10d MI
21d MI
Bright-Field
Birefringence
Tcf21MCM/+; R26eGFP
B
1d MI
2d MI
5d MI
6d MI
10d MI
21d MI
eGFP Col3 Dapi
Infarction / scar area
C
PostnMCM/MCM;R26eGFP
PostnMCM/+;R26eGFP
MI
Age: 8w
9w
10w
tamoxifen
Harvest
D
PostnMCM/+; R26eGFP
PostnMCM/MCM; R26eGFP
GFP Col1 Dapi
GFP Col3 Dapi

Supplemental Figure 4. ECM remodeling post-MI and its effect on αSMA expression in cardiac fibroblasts. (**A**) Representative Picro Sirius Red staining images of heart histological cross-sections taken in bright-field mode or under polarized light for birefringence detection at the indicated time points showing the progression of fibrosis and collagen maturation after MI injury. Scale bars = 1 mm. (**B**) Representative IHC heart images showing morphological changes in Col3 (red) along with *Tcf21* lineage-traced (eGFP^{+}) fibroblasts within the MI region from *Tcf21*$^{MCM/+}$;*R26*^{eGFP} mice at the indicated time points. Dapi shows nuclei (blue). Scale bars = 20 μm. (**C**) Experimental scheme of tamoxifen treatment to *Postn*$^{MCM/MCM}$;*R26*^{eGFP} and *Postn*$^{MCM/+}$;*R26*^{eGFP} mice from day 2 to day 3 post-MI by daily IP injections. Hearts were then harvested at 2 weeks post-MI. (**D**) Representative IHC heart images showing Col1 (red, upper panels), Col3 (red, lower panels) and *Postn* lineage-traced (eGFP^{+}) fibroblasts in the infarct region of hearts from *Postn*$^{MCM/+}$;*R26*^{eGFP} mice and *Postn*$^{MCM/MCM}$;*R26*^{eGFP} mice 2 weeks after MI. Nuclei are shown with Dapi (blue). Scale bars = 20 μm.

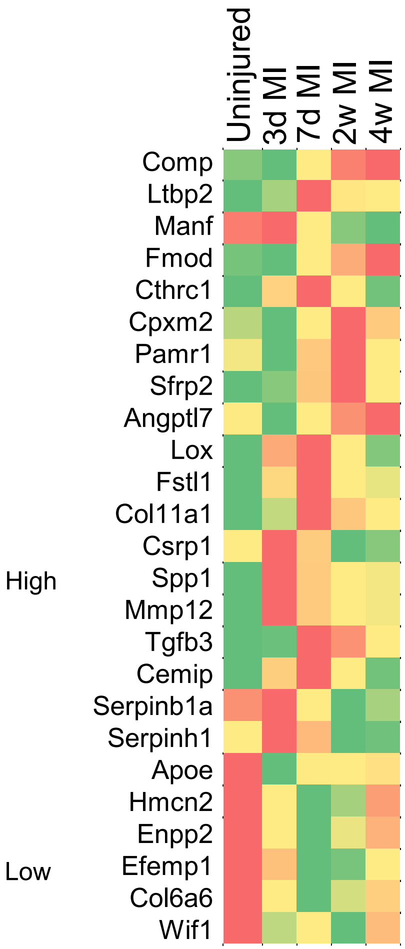

Supplemental Figure 5. Listing of differentially expressed secreted factors from the mRNA sequencing analysis as described in Figure 9. The listed genes all have signal sequences although some are known ECM encoding genes while others are bonafide secreted signaling effectors.

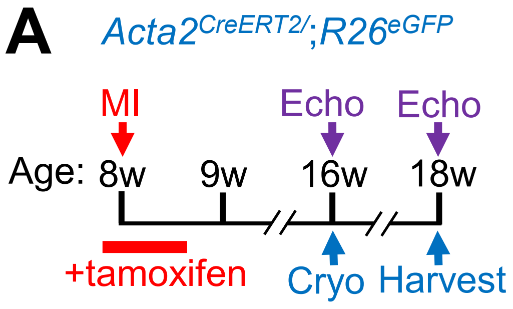

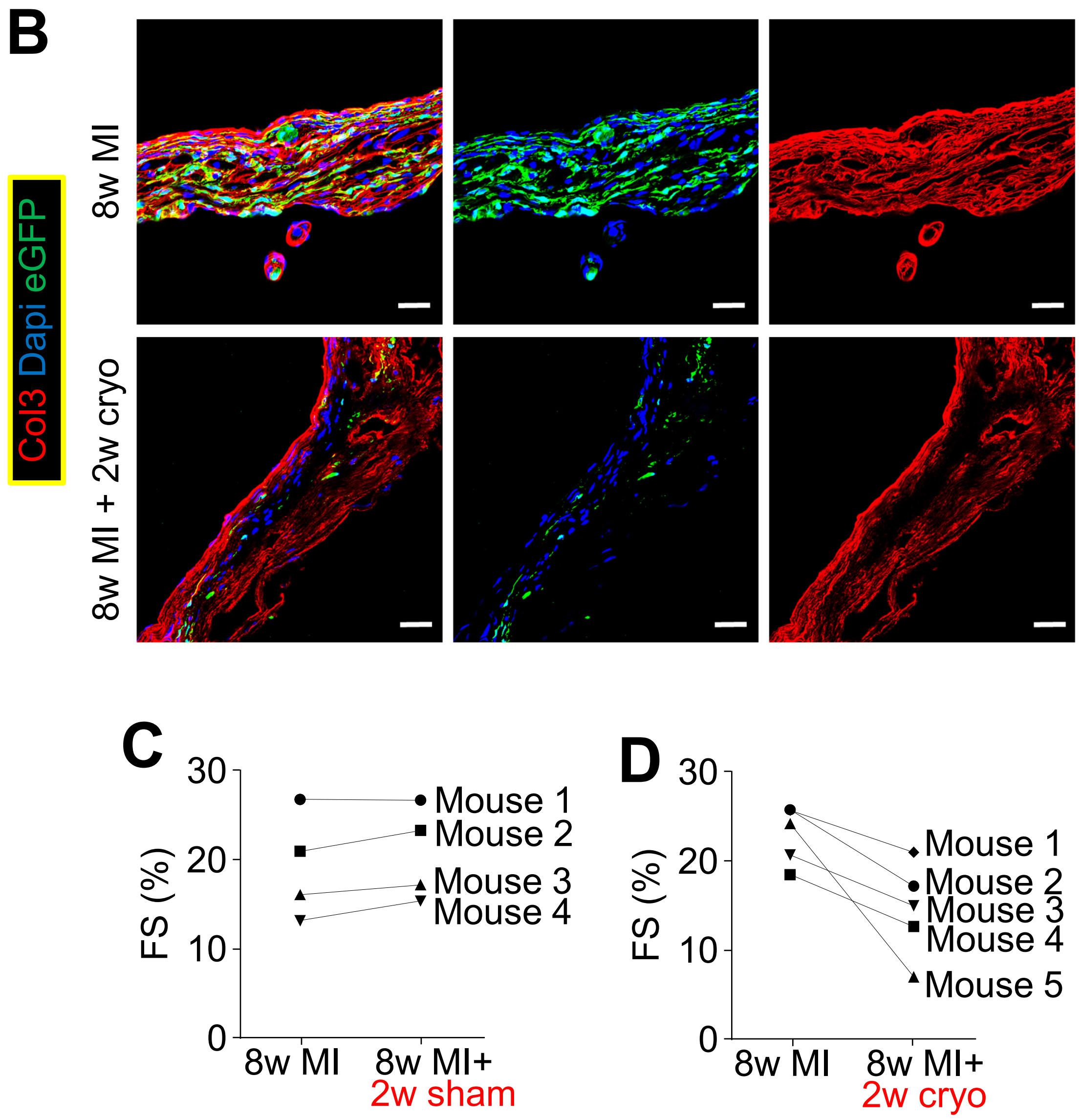

Supplemental Figure 6. Secondary cryoinfarction of MI scar area to kill matrifibrocytes. (**A**) Experimental scheme whereby *Acta2*$^{CreERT2/}$;*R26*^{eGFP} mice were given tamoxifen through daily IP injections from day -1 to day 4 post-MI. Mice were subjected to MI and allowed to recover for 8 weeks. There-after the MI scar area was subject to cryoinjury or sham operation. Hearts were harvested 2 weeks after cryoinjury for IHC analysis. Echocardiography was performed before cryoinjury and 2 weeks after cryoinjury. (**B**) Representative IHC images showing αSMA lineage-traced (eGFP^{+}) fibroblasts and Col3 content (red) in the infarct region at 2 weeks after cryoinjury or sham operation performed at 8 weeks post-MI. Nuclei are shown with Dapi (blue). Scale bar: 20 μm. (**C**) Fractional shortening percentage (FS%) of hearts of mice at 8 weeks post-MI and 2 weeks after sham operation, or (**D**) 2 weeks after cryoinjury.

Supplemental Table

	Unregulated genes, 4W vs uninjured		Downregulated genes, 4W vs uninjured	
	Biological processes	*p*-value	Biological processes	*p*-value
ECM modification, and Bone and cartilage development	Cartilage development	5.50e-11	Positive regulation of osteoblast differentiation	8.50e-05
	Chondrocyte differentiation	3.70e-10	Extracellular matrix organization	3.80e-08
	Bone development	3.20e-08		
	Osteoblast differentiation	6.30e-05		
	Negative regulation of ossification	6.70e-08		
	Extracellular matrix organization	7.90e-09		
	Connective tissue development	3.10e-09		
Cell communication and signaling	Cell adhesion	2.30e-15	Cell adhesion	1.00E-14
	Cell junction organization	5.80e-04	Positive regulation of cell-substrate adhesion	2.10e-06
	Regulation of intracellular signal transduction	6.50e-10	Cell junction organization	1.10e-05
	Negative regulation of cell communication	4.90e-14	Positive regulation of intracellular signal transduction	1.30e-11
	Response to endogenous stimulus	5.50e-15	Positive regulation of protein phosphorylation	1.80e-12
	Negative regulation of cellular response to growth factor stimulus	2.30e-08	Positive regulation of transmembrane receptor protein serine/threonine kinase signaling pathway	4.40e-04
	Regulation of protein phosphorylation	4.30e-08	Positive regulation of response to external stimulus	8.40e-12
	Negative regulation of cellular response to TGFb stimulus	4.60e-05	Positive regulation of cell communication	2.40e-15
	Negative regulation of transmembrane receptor protein serine/threonine kinase signaling pathway	4.20e-07		
	Positive regulation of MAP kinase activity	1.30e-05		
	Positive regulation of protein kinase B signaling	5.90e-04		
Cell death	Negative regulation of cell death	1.20e-08	Positive regulation of cell death	5.30e-07
	Negative regulation of apoptotic process	6.00e-06	Positive regulation of apoptotic process	2.10e-07
Cytoskeleton organization	Actomyosin structure organization	8.30e-07	Positive regulation of cytoskeleton organization	0.029
	Regulation of cell projection organization	1.60e-06		
	Actin filament-based process	2.60e-14		
	Actin cytoskeleton organization	1.40e-13		
	Regulation of actin filament bundle assembly	6.10e-07		
Cell motility and proliferat ion			Positive regulation of cell migration	5.30e-14
			Positive regulation of cell motility	4.40e-14
			Positive regulation of cell proliferation	2.00e-10

Parent biologic ontology processes are shown in the left column and the specific child biologic sub-processes are shown in the 2 columns listed as "biologic processes" (up or down). The data were assembled from mRNA microarray analysis of *Tcf21* lineage-traced cardiac fibroblasts from "uninjured" heart versus fibroblasts isolated from the mouse MI scar area 4 weeks after injury. *P* values show significance of the relationship with the biologic process. n=3 for uninjured and n=3 for 4W MI.

Table shows parent biological processes most significantly changed between *Tcf21* lineage-traced fibroblasts isolated from the uninjured heart and those from the infarct area of hearts 4 weeks post-MI and the caculated *p*-value (Assayed with Affymetrix microarrays). Data analyzed using all differentially expressed (DE) genes, upregulated genes, and downregulated genes are presented separately in different columns. (**B**) Table shows selected individual child biological processes that are important in fibroblast physiology and are significantly changed between *Tcf21* lineage-traced fibroblasts isolated from the uninjured heart and those from the infarct area of hearts 4 weeks post-MI and the calculated *p*-value (Assayed with Affymetrix microarrays). Upregulated and downregulated genes were analyzed and presented separately. n=3 (uninjured, 3D MI, 7D MI, and 4W MI), n=2 (2W MI).
